# The Influence of the Central Metal (Zn) in the Porphyrin Skeleton on the Mechanism Induced by Photodynamic Therapy

**DOI:** 10.3390/cancers18162567

**Published:** 2026-08-10

**Authors:** Rostyslav Marunych, Dorota Bartusik-Aebisher, Barbara Smolak, Klaudia Dynarowicz, David Aebisher

**Affiliations:** 1 Doctoral School, Faculty of Medicine, Collegium Medicum, University of Rzeszów, 35-310 Rzeszow, Poland; rostyslavm@dokt.ur.edu.pl; 2 Department of Biochemistry and General Chemistry, Faculty of Medicine, Collegium Medicum, University of Rzeszów, 35-310 Rzeszow, Poland; dbartusikaebisher@ur.edu.pl (D.B.-A.); kdynarowicz@ur.edu.pl (K.D.); 3 Department of Diagnostic Imaging and Nuclear Medicine, Faculty of Medicine, University of Rzeszów, 35-310 Rzeszow, Poland; bsmolak@ur.edu.pl; 4 Department of Photomedicine and Physical Chemistry, Faculty of Medicine, Collegium Medicum, University of Rzeszow, 35-310 Rzeszow, Poland

**Keywords:** photodynamic therapy, Zn(II) porphyrins, metalloporphyrin, singlet oxygen, ROS, theragnostic, subcellular localization

## Abstract

This review analyzes how Zinc(II) coordination alters the electronic configuration of porphyrin-based photosensitizers to optimize reactive oxygen species (ROS) generation and subcellular targeting in photodynamic therapy (PDT). By focusing on the structural design principles that govern excited-state behavior, the work moves beyond clinical descriptions to provide a mechanistic understanding of how engineered metalloporphyrins can achieve precise tumor destruction. When these engineered metal porphyrins are exposed to specific wavelengths of light, they transfer energy to create ROS, such as singlet oxygen, which directly damages and kills tumor tissue. The review evaluates structural modifications that drive selective accumulation within critical subcellular organelles, notably the mitochondria, to maximize cytotoxic efficiency. By analyzing the impact of the tumor microenvironment on hypoxia, the work outlines strategies for maintaining efficacy in oxygen-deprived zones and highlights how the biocompatible, redox-inactive nature of Zinc(II) minimizes systemic toxicity, providing a blueprint for the design of targeted, translation-ready photosensitizers.

## Simple Summary

Zinc(II) coordination with porphyrin-based photosensitizers optimizes photodynamic therapy by enhancing reactive oxygen species generation, improving mitochondrial targeting, and maintaining efficacy in hypoxic tumor environments. These engineered metalloporphyrins minimize systemic toxicity due to the biocompatible nature of Zinc(II), providing a strategic design for effective, targeted cancer treatment.

## 1. Introduction

### 1.1. Photodynamic Therapy Is a Mechanism-Oriented Therapeutic Strategy

**Figure 1:**
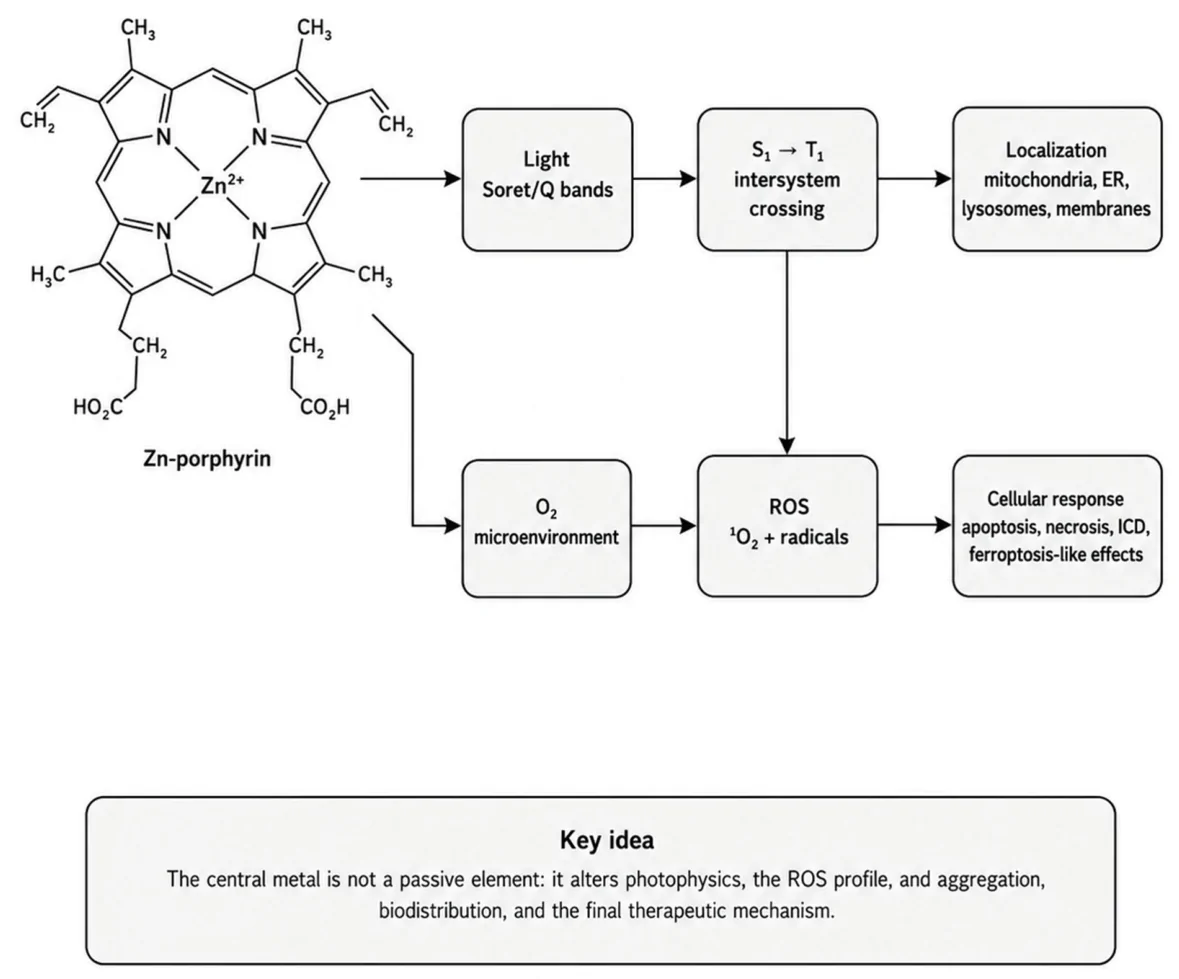
The characteristics of Zn-porphyrin.

Photodynamic therapy (PDT) belongs to the locally managed photochemistry approaches. The therapeutic effects appear only if three components are present: a photosensitizer, light with the appropriate wavelength and molecular oxygen [1,2,3,4]. This three-component nature principally differentiates PDT from a large number of pharmacological interventions. The photosensitizer can be localized in the tissue without determined dark toxicity and cytotoxic influence. Cytotoxic influence must form only after light exposure. Due to this, PDT is presented not as a simple “light curing”, but as a controlled biophysical process in which the absorption of the photon starts a cascade of electrical, photochemical, and redox cell responses [1,2,3]. Despite the clinical success of PDT as a minimally invasive oncological intervention, contemporary photosensitizers encounter persistent limitations including poor tissue penetration, off-target phototoxicity, and severely diminished efficacy within hypoxic tumor environments. While recent technological developments utilize advanced molecular engineering, nanotechnology, and oxygen-independent pathways to bypass these barriers, a stronger chemical framework is required to optimize therapeutic performance. Zinc(II)-porphyrins offer a uniquely balanced alternative due to their distinct d^10^ electronic configuration, which introduces a moderate heavy-atom effect that accelerates intersystem crossing without quenching the singlet state. This specific electronic structure preserves robust fluorescence quantum yields for dual theranostic utility, while the biologically compatible, redox-inactive nature of endogenous Zn(II) minimizes systemic off-target toxicity. The scope of this review encompasses the complete translational pipeline of Zn(II) porphyrins, with the objective of decoding how coordination geometry alters excited-state behavior, evaluating formulation strategies against biological barriers, and providing a rigorous comparative analysis with alternative metalloporphyrins. Rather than offering a purely descriptive survey, this work introduces a unique, mechanism-based design framework that maps exact structural modifications directly to photophysical and biological outcomes, serving as a rational blueprint for next-generation, translation-ready therapeutics (Figure 1).

After absorption of light, the photosensitizer transitions from the ground singlet state S0 to an excited singlet state S1 or another singlet state, which rapidly relaxes to S1. From this state, the molecule can emit fluorescence, lose energy without irradiation, or turn into the triplet state T1 through ISC (ISC) [5,6,7]. Mostly, the long-lived triplet state is key for most therapeutically relevant reactions because it has a long enough lifetime to interact with oxygen, lipids, proteins, or other biological substrates. In the classical mechanism of type II photochemistry, energy transfers from the triplet-state photosensitizer to triplet molecular oxygen with the generation of singlet oxygen ^1^O_2_ [8,9,10]. In the type I mechanism of photochemistry, the observed process is the transfer of electrons from the atom of hydrogen with the generation of a superoxide anion radical, hydrogen peroxide, hydroxyl radicals, and secondary products of lipid oxidation [3,8,11,12].

**Figure 2:**
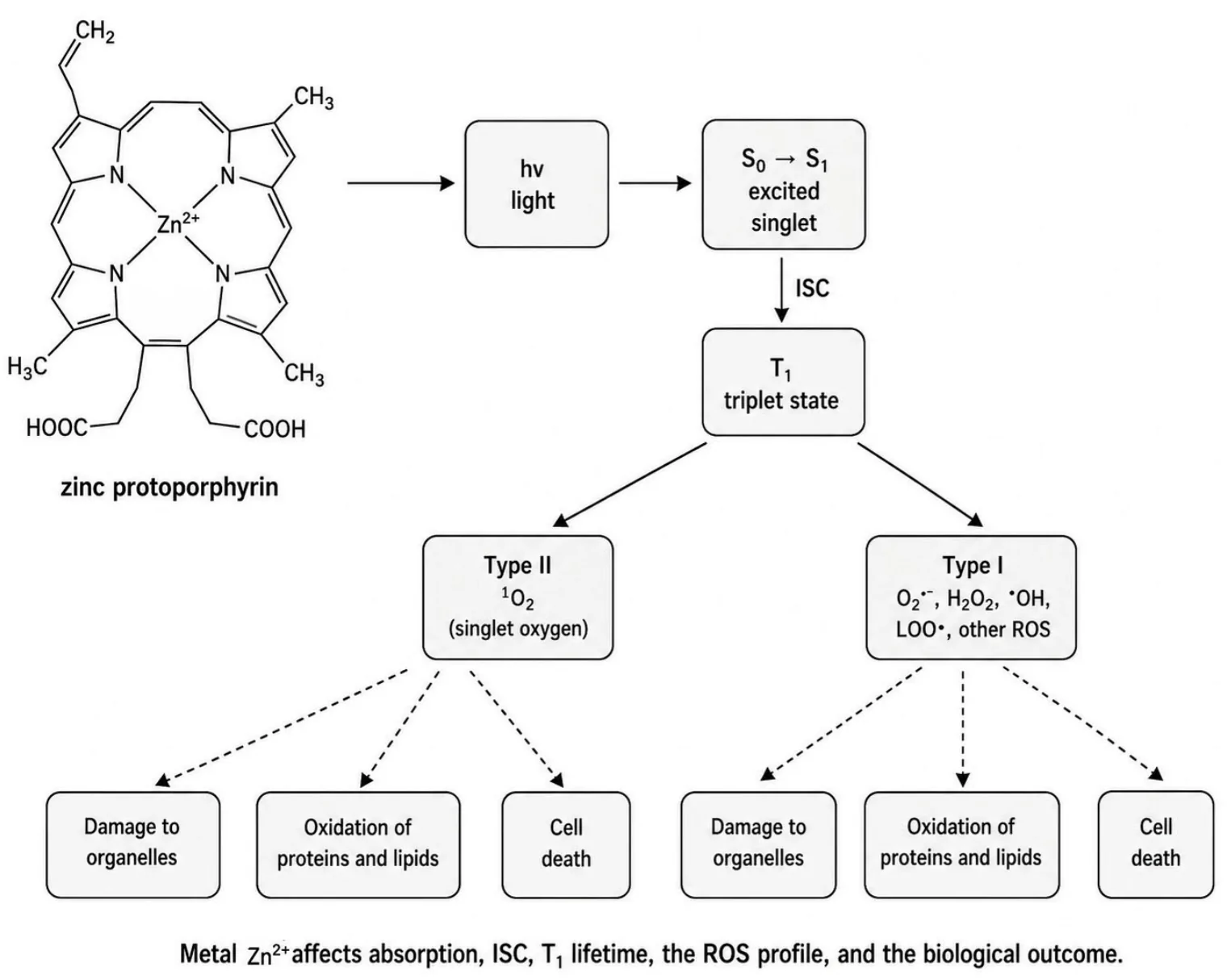
General mechanisms of metalloporphyrin-mediated PDT.

The biological result of PDT depends not only on the amount of ROS (ROS), but also on where they are generated. Singlet oxygen and most radicals have a limited diffusion distance due to their short lifetime in the cell medium. Due to this, the targets of PDT are determined by the subcellular localization of the photosensitizer [8,9,13]. If the compound accumulates in the mitochondria, early consequences can be membrane depolarization, cytochrome C release from the mitochondria, and activation of the internal apoptotic pathway. Lysosomal localization favors permeability of the lysosomal membrane and cathepsin release. The endoplasmic reticulum is affected by the stress caused by unfolded proteins, Ca(II) homeostasis disturbance, and activation of the PERK, IRE1/ERN1 i ATF6-dependent pathways. Damage to the plasma membrane and endothelial cells can, in turn, facilitate necrosis, vascular occlusion, or an inflammatory reaction [3,4,5,14]. The principal photophysical and biochemical sequence of metalloporphyrin-mediated PDT is summarized in Figure 2.

So, PDT is a mechanism-oriented therapeutic strategy, where the dosage of light, the spectral properties of the photosensitizer, oxygen availability, and the cancer microenvironment and the molecular architecture of the photosensitizer form a holistic system [1,2,3,11]. This is very important for porphyrins and metalloporphyrins, because their photochemical properties can be changed not only by peripheral substituents, but also by the central atom in the macrocycle [1,4,5]. The proposed relationship linking Zn(II) coordination to therapeutic outcomes is a simplified conceptual framework designed to highlight the core photophysical cascade, rather than a strictly linear model. It is intended to be expanded into an integrated, multidirectional model where formulation (aggregation, delivery vehicle) and biological factors (oxygen levels, intracellular reductants, subcellular localization) actively modulate every step of the pathway from photoactivation to cell death.

### 1.2. Porphirins as Classical Managed-Modified Photosensitizers

Porphyrins are tetrapyrrole macrocycles, structurally comparable to biological pigments like heme, chlorophylls, and cobalamin systems. Their skeletons consist of four pyrrole rings joined with bridges, forming a long pi-conjugated system [12,15,16]. This electron band corresponds to strong absorption in the Soret peak and less intense peaks in the Q-band in the visible spectrum. For PDT, this has a double meaning: on the one hand, porphyrins efficiently absorb light, and on the other hand, absorption in red and the near-infrared light always needs strengthening for deep tissue penetration [12,15,16].

From a historical point of view, porphyrins or porphyrinoids have become one of the best platforms for the development of PDT. They combine in the same molecule relative chemical stability, the ability to generate ROS, fluorescence visualization, and chemical modifications [12,15,16].

Peripheral phenols, carboxylic acids, sulfates, amines, peptide groups, glycosylated or polymeric fragments can change solubility, charge, amphiphilicity, aggregation, interaction with proteins, and transport into cells. Due to this, the porphyrin scaffold can be tuned into a water-soluble anionic photosensitizer, a membrane cationic compound, a lipophilic mitochondrial agent or a component of a nanoparticle [12,15,16,17].

A key property of porphyrins is the sensitivity of their photodynamic efficacy to their aggregate state. A planar aromatic cycle forms π–π associates easily. It happens mainly in aqueous solution and at higher local concentrations. Aggregation decreases fluorescence, shortens the lifetime of the excited state by half, facilitates non-irradiated relaxation, and decreases the yield of singlet oxygen [12,15,18,19]. Because of this, the modern design of photosensitizers includes not only optimization of the absorption spectrum but also control of the spatial localization of substituent groups and their spatial organization. This includes: the introduction of bulky substituents, ionic or charged groups, axial ligands, polymer covering, liposomes, micelles, or metalloorganic carcasses [12,15,16,19].

The porphyrin platform can be useful for theragnostic applications. Fluorescence allows for evaluating the sensitizer, borders of damaged tissues, subcellular localizations, and photobleaching during the therapeutic processes [12,15,18,19]. But fluorescence and phototoxicity do not always correlate directly. A molecule which can emit a strong fluorescent signal can have a lower triplet state output. Conversely, strong intersystem conversion sometimes decreases the diagnostic signal. The porphyrin photosensitizer must be evaluated as a system in which absorption, fluorescence, triplet dynamics, ROS profile, and localization must be in consensus with one another [6,12,14,16,20].

### 1.3. Why Does the Central Metal Have Matter?

In many PDT, main attention is focused on substituents of porphyrins on their periphery. But the central atom is analyzed as a structural element. The process of inserting the metal into the macrocycle coordination cavity changes the electronic structure of the porphyrin. It changes the symmetry of the molecule, the energy of the triplet and the singlet stages, the probability of intersystem conversion, fluorescence, redox behavior, aggregation and interaction with biomolecules [6,7,14,20].

In different types of metalloporphirins, higher symmetry simplifies Q-bands. And the metal nature determines whether an excited state is directed toward irradiation, productive generation of ROS, or, depending on the structure, quick non-irradiation deactivation [6,7,14,20].

The central metal has an influence on PDT through a minimum of four interconnected levels. The first level is photospectral: the metal can change the absorption band, molar coefficient of extinction, and modify the intensity of fluorescence. The second level is photodynamical: spin-orbit interaction, diamagnetic or paramagnetic character of the metals influence on the generation of the singlet oxygen. The third level is chemical: Fe, Mn, and Cu can have an influence on antioxidant properties [6,7,14,20,21]. The fourth level is biological: the charge, planarity, axial coordination, protein interactions and aggregation of the metalloporphirin determine its transport in the cell and subcellular localization [6,7,14,15,16,17,18,19].

Zn(II)-porphyrins occupy a special place among metalloporphyrin photosensitizers. The ion of Zn(II) has a d10 electronic configuration, is diamagnetic under physiological conditions, and does not provide redox cycling [6,14,20]. Because of this, the Zn-center can change the photophysics of porphyrin without expressed changes in metal-catalyzed dark toxicity. There are a lot of cases when Zn(II)-porphyrins demonstrate a favorable combination of fluorescence, triplet stage formation, and generation of singlet oxygen, which makes it favorable for visualization of PDT also [6,14,17,21,22]. At the same time, Zn(II) is not a universal amplifier. Really, effectiveness depends on the ligand surrounding, peripheral groups, aggregation, solvent, presence of protein, and formulation [6,14,17,22].

Comparison with another method demonstrates that metallation does not guarantee improvement of PDT. Pd(II) and Pt(II) can amplify intersystem conversion through the heavy-atom effect and form a long-term triplite stage, but with a lower fluorescent signal. Mg(II) porphyrins biologically associate with chlorophyll systems, but can be less stable in acidic media. Sn(IV) and Al(III) form axially coordinated complexes, which have an influence on amphiphilicity and aggregation. Fe(III), Mn(III), Cu(II) controversially, favor deactivation of existed states, or transmit photodynamic response in difficult redox-modulation [6,7,14,20,21]. Therefore, the central metal is an active regulation mechanism, but not a passive in the center of the macrocycle [6,7,13].

### 1.4. The Goal and Constraints of the Review

The goal of this review is to systematically analyze how the coordination of the central metal in the porphyrin ring can change the mechanisms of PDT at the molecular, organelle, cell, and translational levels.

Main attention is focused on Zn(II) porphyrins as models, which have a central metal that is redox-inert in biological media. But its behavior has an influence on metalloporphyrin structure, fluorescence, triplet dynamics, generation of singlet oxygen, and biodistribution. This focus allows separating the photophysical role of the metal from effective direct metal-catalyzed redox cycling, which is typical for some Fe, Mn, Cu-porphyrins [6,7,14,20,22].

The review is built around a central theme: the ion of the metal in the porphyrin is a neutral structure, but has an influence on the structural, photophysical, and localization responses in PDT along a unified axis. And in the next chapters, we will analyze the chemical basis of metalloporphyrin photosensitizers, the influence of metals on absorption, fluorescence, intersystem crossing, and triplet states, the balance between type I and type II mechanisms, subcellular localization, biochemical damage to proteins, lipids, and organelles (mitochondria, endoplasmic reticulum, and chromosomes), and the activation of cell death pathways. Separate chapters will be dedicated to the comparison of Zn(II) porphyrins and non-metal porphyrins and other metal porphyrins, and their influence on the cancer microenvironment, nanoformulation, experimental methods and clinical translation perspectives [6,7,12,14,16,19].

This review does not claim to be a full catalog of all synthesized Zn(II) porphyrins. Instead, the task of this publication is a mechanistic generalization: how properties of one central metal can influence PDT; under which conditions Zn(II) provides superiority; when peripheral chemistry or formulation becomes decisive; and how the porphyrin macrocycle can induce different biological responses depending on the metal, localization, and oxygen context. This perspective is important for the rational design of new-generation photosensitizers, in which photodiagnostics, control of ROS profiles, organelle targeting, immunogenic response, and pharmacokinetic properties are analyzed as parameters, but not as independent characteristics [2,12,16,19,23].

## 2. Structure and Chemical Basis of Metallophyrins Photosensitizers

### 2.1. Main Structure of Porphyritic Macrocycle

Porphyrins are one of the most important platforms for PDT because they are connected to a higher ability to absorb light, with the ability to be chemically modified and biologically recognized. In the simplest definition, porphyrin consists of four pyrrole rings, joined with methylene bridges in the flat or almost flat tetrapyrrole microcycle. This electron system maintains the conversion of molecules to the excited singlet state after photon absorption, and subsequently, the formation of the triplet state, from which electron or energy transfer reactions occur [6,24,25,26].

The internal cavity of the macrocycle is created by four nitrogen atoms. In the free base of porphyrins, two of them are free and bear protons, while another two have a pyridine-like nature and can coordinate a cation of the metal. This makes porphyrins not only a chromophore but a ligand with a clearly determined coordination center. Insertion of a metal changes molecular symmetry. It changes the distribution of the electron density, acid-base properties of the nitrogen atoms, conformational rigidity, and ability to form axial coordination. So, the central cavity is not a passive volume. It is a chemical node that can manage the photobiological behavior of the molecules [7,24,25,26].

Peripheral substituents on meso- and beta-positions determine solubility, charge, amphiphilicity, aggregation, and interaction with plasma proteins, membrane transport, and intracellular localization. But their effect should not be studied separately against the central metal atom. The same composition of peripheral groups can give a different profile upon metallation because spectral characteristics, fluorescence quantum yield, triplet outcome, time of life of the excited states, and tendency to self-assemble all change. Due to this, the photosensitizer structure must be analyzed as an integrated system: macrocycle—central metal—peripheral groups—formulation—biological microenvironment [25,26,27,28,29].

### 2.2. Metal Insertion in Porphyrinic Nuclear

Porphyrins metallation is a coordination reaction in which the cation of the metal substitutes two of the internal protons and binds with four coordinated atoms in the nitrogen in the macrocycle. For photodynamic therapy, the principal meaning is not only in metallation but also in the ionic radius of the metal, oxidation state, electron configuration, and coordination number, geometry of the complex, and strength of the Me-N bond. Metals with a size close to the optimal size for the porphyrin cavity mostly create more planar complexes. If the cation is too large or needs a large coordination number, the macrocycle can deform, and the metal moves out of the plane of the porphyrin. Such deformations can have an influence on the overlapping π-orbitals, energy of excited states, and interactions with molecular oxygen [7,30,31,32].

Coordination geometry is extremely important for the biological behavior of metalloporphyrins. Four-coordinated complexes, typical for most Zn(II) porphyrins, can stay flat. But Zn(II) can bind axial ligands, for example, a molecule of water, imidazole, a histidine group, amines, or natural donor groups of biomolecules. Axial coordination changes solubility, aggregation, protein binding, and orientation of molecules in the membrane. For metals with a higher degree of oxidation, like Al(III), Sn(IV), and In(III), axial ligands can be integral parts and can facilitate hydrophilicity or maintenance of target delivery [7,22,30,31,32].

The strength of the metal-nitrogen bond determines the stability of the photosensitizer in biological media. A labile metal can be set free or exchanged for another cation. This can cause loss of potential photochemical properties and increase potential toxicity. A metal that is too inert can maintain too high stability. But sometimes quenching of excited states can occur. Due to this, the optimal metal for PDT is not simply the metal that fits the coordination cavity. This is the metal that maintains the necessary balance between stability, photogeneration of active forms of oxygen, low dark toxicity, and controlled biodistribution [7,22,30,31,32,33].

It is necessary to review Zn(II) separately because it demonstrates coordination influence on the macrocycle and no relevant redox activity once general principles of metallation have been reviewed.

### 2.3. Zinc as a Central Metal

Zn(II) occupies a special place among the central metals of porphyrin photosensitizers. Its d10 electron configuration indicates a filled d-shell; therefore, under typical physiological conditions, Zn(II) does not participate in the intense single-electron redox cycling characteristic of Fe, Cu or Mn complexes. This is important for PDT, as Zn(II) porphyrins can act primarily as photophysical generators of excited states and singlet oxygen, rather than as uncontrolled catalysts of redox processes. Such redox inertness enhances the predictability of the mechanism, although it does not eliminate the influence of peripheral substituents, the medium, aggregation and localization [7,30,31,32].

Zinc metallation of porphyrin typically increases the symmetry of the macrocycle compared with the free base. Spectroscopically, this often manifests as a simplification of the Q-region and changes in the intensity of individual bands. Zn(II) can also enhance fluorescence and support the efficient formation of the triplet state, making Zn-porphyrins suitable not only for therapy but also for photodiagnostics and delivery monitoring. This is one of the reasons why Zn(II) porphyrins are regarded as theranostic platforms: a single molecular system can provide optical imaging, local photoactivation and assessment of biodistribution [6,17,22,24,30].

Zn(II) coordination chemistry also offers opportunities for regulating aggregation. Aggregation is one of the main causes of reduced photodynamic efficiency in porphyrins, as π–π-stacking promotes self-quenching, reduces the accessibility of the triplet state to oxygen and alters intracellular transport. The presence of Zn(II) and potential axial ligands may reduce or reorient aggregation if the molecule possesses suitable amphiphilicity or is incorporated into a nanoformulation. On the other hand, the flatness of the Zn-porphyrin core may promote stacking if the peripheral architecture does not prevent the macrocycles from coming into close proximity. Therefore, Zn(II) alone does not guarantee anti-aggregation properties; it provides a chemical framework that must be combined with a rational design of substituents and the carrier [17,22,25,28,29,34].

In a biological context, Zn(II) porphyrins can interact with albumin, lipoproteins, membranes, nucleic acids and proteins containing donor groups for axial coordination. These interactions are not necessarily receptor-specific, but they can determine circulation time, cellular uptake, intracellular localization and phototoxicity. It is particularly important that Zn(II) porphyrins often demonstrate a high capacity for generating singlet oxygen, but the ultimate biological effect depends on exactly where in the cell this short-lived oxidant is formed. Therefore, the structural chemistry of Zn-porphyrins is directly linked to issues of localization, organelle damage and the choice of cell death pathway [6,22,26,28,31,35,36].

To assess the specificity of Zn(II), its properties should be compared with the behavior of other metals, which can alter the photophysics and biochemistry of porphyrins in various ways.

### 2.4. Comparison with Other Central Metals

A comparison of Zn(II) with other metals shows that the central ion can either enhance or limit PDT activity. Mg(II)-porphyrin and chlorophyll-like systems are biologically relevant, as magnesium is the central metal in chlorophyll; however, such complexes may have limitations in terms of stability and photochemical controllability under therapeutic conditions. Al(III), Sn(IV) and In(III) form complexes with axial ligands, which are useful for enhancing water solubility, regulating charge and creating conjugates. In such systems, the central metal influences not only the photonic properties of the macrocycle but also the three-dimensional shape of the molecule and its interaction with biological barriers [7,30,31,32].

Pd(II) and Pt(II) are examples of metals in which the heavy-atom effect is significant. Enhanced spin–orbit coupling can significantly increase ISC and triplet yield. This is beneficial for the generation of ROS, but excessive enhancement of non-radiative relaxation or changes in redox behavior may reduce the diagnostic value of fluorescence. Some Pd-porphyrins exhibit very high triplet yields and pronounced phototoxicity, making them promising for PDT and the photodynamic inactivation of microorganisms. At the same time, such metals require careful assessment of dark toxicity, pharmacokinetics and long-term safety [30,32,33,37,38].

Fe(III), Mn(III) and Cu(II) porphyrins constitute a separate group, as the central metal can participate directly in redox processes. Depending on their structure and conditions, such complexes can act as photosensitizers, catalysts for the conversion of ROS, modulators of oxidative stress, or even antioxidant-like compounds. For example, Mn porphyrins are often considered in the context of mimicking superoxide dismutase activity, while Fe and Cu complexes may be involved in reactions similar to the Fenton reaction or in electron transfer. For PDT, this creates a duality: a redox-active metal may enhance the type I component and be beneficial under hypoxia, but may also contribute to unpredictable side reactions or the quenching of the excited state [7,22,30,32,39,40].

Metal-free porphyrins remain an important point of comparison. Their inner cavity contains two protons, so the molecule has lower symmetry and four characteristic Q-bands. They can be effective photosensitizers, but metallation often allows for targeted modification of the spectrum, fluorescence, triplet yield, photostability and biological binding. At the same time, metallation is not automatically beneficial: some metals, particularly those with an unfavorable electronic configuration or excessive redox activity, can quench excited states and reduce the photodynamic effect. Thus, the central metal should be regarded as a key structure-activity parameter, on a par with peripheral substituents and the delivery system [6,24,30,32,34].

From a practical standpoint, an optimal central metal should provide strong absorption in the therapeutically relevant region, sufficient triplet yield, high or controllable generation of ROS, low dark toxicity, chemical stability, minimal aggregation and compatibility with formulations. Zn(II) meets many of these criteria due to its redox inertness, good compatibility with the porphyrin core and its ability to support fluorescent and photodynamic properties. However, it is not a one-size-fits-all solution: in hypoxic tumors, bacterial biofilms or combined systems, metals that more strongly support type I pathways or possess additional catalytic functions may be more useful. This is precisely why the rational design of a metal porphyrins photosensitizer should begin with the selection of the central metal in accordance with the desired PDT mechanism, rather than merely modifying the periphery of the macrocycle [7,22,25,30,35,38,41].

A brief summary of the structural parameters through which the central metal modifies the PDT activity of the porphyrin is provided below [6,7,30,32,33].

Structural differences between metalloporphyrins are of functional importance primarily because they alter photophysical events following light absorption.

## 3. The Effect of the Central Metal on the Photophysics of Porphyrins

The photophysics of porphyrin photosensitizers determines whether the absorbed light energy will be converted into a therapeutically useful flux of ROS or lost through fluorescence, internal conversion, thermal relaxation, photobleaching, or aggregation-induced quenching. The central metal in the porphyrin core directly alters the electronic structure of the macrocycle, the symmetry of the molecule, the energy levels of the singlet and triplet states, the strength of the spin–orbit coupling, and the probability of an intersystem transition. Therefore, metallation is not a secondary synthetic modification: it transforms porphyrin from an organic chromophore into a coordination photochemical system in which the metal, macrocycle, peripheral substituents, solvent, state of aggregation, and biological microenvironment form a unified mechanistic framework for PDT [42,43,44,45]. For Zn(II) porphyrins, this role is particularly typical. The Zn(II) ion has a d10 electron configuration and does not normally participate in direct biological redox cycling, but it is capable of enhancing the organization of the porphyrin chromophore, altering absorption spectra, sustaining significant fluorescence, and promoting the formation of triplet states suitable for singlet oxygen generation [6,17,42,43,44,46,47].

It is advisable to begin a photophysical analysis with absorption spectra, since they determine whether a photosensitizer can be activated by therapeutic light.

### 3.1. Absorption Spectra: Soret and Q Bands After Metallization

The porphyrin macrocycle exhibits an intense Soret band in the blue-violet region and a set of less intense Q-bands in the visible region. This spectral profile stems from a delocalized π-electron system, which is often described within the framework of Gouterman’s four-orbital model: two occupied orbitals of similar energy and two unoccupied orbitals of similar energy form the main electronic transitions of porphyrin [42,43]. In free-base porphyrins, two internal protons break the higher symmetry of the macrocycle; therefore the Q-spectrum often contains four bands. After a metal is introduced into the central coordination cavity, the symmetry increases, and the Q-spectrum is usually simplified to two more intense and better-defined bands. This is not only an analytical marker of metallation but also a functional parameter of PDT, since the spectral position of the Q-bands determines how effectively the photosensitizer can be activated by light capable of penetrating tissues [42,45,46].

Zn(II) itself does not cause low-energy d–d transitions, so the spectrum remains largely ligand-centered and is associated with π–π* transitions in the macrocycle. However, the coordination of Zn(II) alters the planarity, the electron density on the nitrogen atoms, and the chromophore’s sensitivity to axial ligands. Pyridine, imidazole, carboxylate, or protein donors can be temporarily coordinated to Zn(II), altering the microenvironment and spectral properties of the molecule. In biological systems, this means that the same Zn-porphyrin can possess different photophysical characteristics in an organic solvent, a lipid membrane, an albumin complex, a nanoparticle, or within an organelle [6,10,17,43,46,47,48].

After analyzing the absorption spectrum, it is important to determine how the absorbed energy is distributed among fluorescence, non-radiative relaxation, and subsequent photochemical pathways.

### 3.2. Excited Singlet States, Fluorescence, and Diagnostic Potential

Upon photon absorption, the porphyrin molecule undergoes a transition to an excited singlet state. It is important to clarify that an individual molecule cannot split its excitation energy among different relaxation pathways. The reported percentages for fluorescence and ISC are probabilities observed across a large population of molecules. Upon absorbing a photon, a single molecule must select a single, discrete quantum path to return to the ground state. For PDT, it is not the maximum fluorescence itself that is important, but rather the balance between diagnostic visibility and photochemical productivity. Excessively high fluorescence quantum efficiency can compete with triplet formation, whereas overly rapid non-radiative relaxation reduces both fluorescence imaging and the generation of ROS [17,44,45,47].

Zn(II) porphyrins often occupy an intermediate and practically useful position. They can demonstrate sufficiently intense fluorescence for localization studies, monitoring of cellular accumulation, and potential theranostic applications, while retaining the ability to undergo efficient ISC. While open-shell Cu(II)-, Fe(III)-, and Mn(III)-porphyrins undergo rapid non-radiative quenching mediated by metal-centered or charge-transfer states, closed-shell Zn(II) analogs exhibit significantly less pronounced suppression of ligand-centered fluorescence [46,49,50]. This is of direct relevance for image-guided PDT: the photosensitizer must not only destroy cells upon illumination but also indicate where it has accumulated prior to irradiation. Therefore, Zn-porphyrin systems are often considered platforms that combine therapeutic and diagnostic modules [6,47,48].

However, the fluorescence signal should not be equated with photodynamic efficiency. Within a cell, it depends on concentration, pH, medium viscosity, protein binding, membrane intercalation, and the degree of aggregation. For example, bright fluorescence in the lipid phase may indicate good accumulation in membranes but does not guarantee effective contact with molecular oxygen or critical biological targets. Conversely, a moderate fluorescence signal in mitochondria or the endoplasmic reticulum may have a stronger biological effect if the photosensitizer is located near proteins, lipids, and calcium systems that are sensitive to oxidation [47,48,49,50,51].

Since fluorescence is only one of the possible deactivation pathways, the next key step is to analyze the ISC and the formation of the triplet state.

### 3.3. ISC and Formation of a Triplet State

The key event in PDT is the conversion of the excited singlet state of the photosensitizer into a long-lived triplet state. It is the triplet state that has a sufficiently long lifetime to interact with molecular oxygen or biological substrates. The central metal affects this process through spin–orbit coupling, a change in the energy gap between the singlet and triplet levels, the emergence of charge transfer states, and possible metal-centered relaxation channels [10,43,44,52].

Heavy metals, such as Pd(II) and Pt(II), can dramatically enhance ISC due to the heavy-atom effect; therefore, their porphyrin complexes often feature high triplet yields and long-lived phosphorescent or triplet states. This is useful for photosensitization but may be accompanied by increased dark-state toxicity, more complex pharmacokinetics, or other biocompatibility issues [32,52,53]. Zn(II) is not as strong a “heavy-atom” center, but as a diamagnetic d10 metal, it is capable of enhancing the structural order of the macrocycle and supporting efficient ligand-centered triplet formation without direct redox complications. Therefore, Zn-porphyrins are often viewed as a compromise between fluorescent viability, high photochemical activity, and relative biocompatibility [10,32,49].

In contrast to Cu(II), Fe(III), and Mn(III) porphyrins possess unpaired d-electrons or accessible metal-centered states, which can accelerate internal conversion, quench excited states, or shift the photochemistry into a redox-catalytic regime. In such complexes, low phototoxicity does not always imply weak absorption; sometimes the chromophore absorbs light well, but the energy is rapidly lost before a productive interaction with oxygen can occur. Thus, for PDT, not only the spectral absorption coefficients but also the kinetic fraction of the excited state are important [32,49,50].

The formation of a triplet state alone does not guarantee effective PDT; its energy, lifetime, and ability to interact with oxygen become decisive factors.

### 3.4. Triplet State Lifetime, Triplet Energy, and Oxygen Sensitization

For a Type II mechanism, the energy of the photosensitizer’s triplet state must be sufficient to convert the triplet ground state of oxygen into singlet oxygen. In addition to the energy match, the T1 lifetime must be sufficiently long for the photosensitizer to encounter O_2_ through diffusion. An excessively short triplet state lifetime leads to premature energy dissipation before oxygen transfer can occur. Meanwhile, a long-lived triplet state operating in an oxygen-poor environment similarly limits photochemical efficiency [44,45,52,54].

Zn(II) porphyrins often exhibit a favorable combination of triplet energy, triplet yield, and the ability to generate ^1^O_2_. This explains why many Zn-porphyrin derivatives, including dimers, amphiphilic systems, and porphyrin nanostructures, are being investigated as singlet oxygen generators for classical and two-photon PDT [17,47,55]. Nevertheless, the numerical value of the quantum yield of singlet oxygen is not a universal constant for a molecule. It depends on the solvent, the presence of aggregation, the oxygen regime, the excitation wavelength, the concentration, the measurement method, and the reference standard. Therefore, data obtained in chloroform or dimethylformamide cannot be directly extrapolated to a cell or tumor [6,52,55].

**Figure 3:**
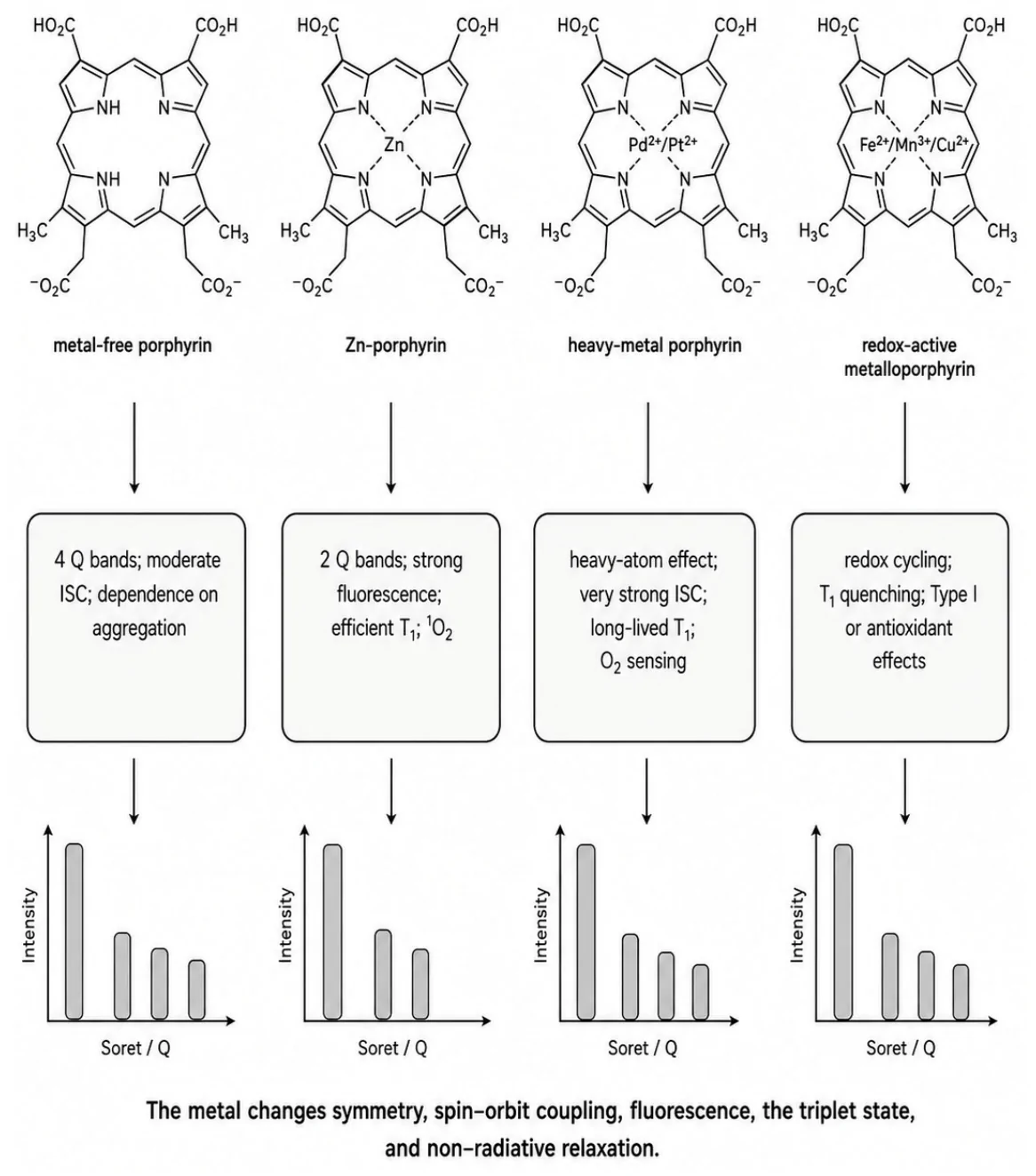
Influence of central metal on porphyrin photophysics. At the bottom of Figure 2, on the X-axis of each subgraph are representative bars presenting electronic absorption bands. Tall leftmost bar is Soret Band. This is the strongest absorption band. In terms of wavenumbers, it appears in the high-energy region around 23,000 to 26,000 cm^−1^ (corresponding to a wavelength of 380–430 nm in the blue/UV spectrum). Shorter cluster of bars to the right is Q Bands. These are weaker absorption bands. In terms of wavenumbers, they appear in the lower-energy region spanning 14,000–20,000 cm^−1^ (corresponding to a wavelength of 500–700 nm in the visible yellow/red spectrum).

In a biological environment, the triplet state is not merely a precursor to singlet oxygen. It can also participate in the transfer of an electron or a hydrogen atom, especially if reducing agents, lipids, protein residues, or membrane components are present. Thus, even Zn-porphyrins, which in simple solutions behave primarily as type II photosensitizers, can partially contribute to type I photodynamic effects within cells. The decisive factors are the local oxygen concentration, the electron donor properties of the microenvironment, aggregation, and the distance to the biological target [53,54]. The principal effects of different classes of central metals on porphyrin symmetry, spectral structure, fluorescence and triplet-state formation are compared in Figure 3.

After considering productive excited states, it is also necessary to take into account the opposite process—aggregation-induced quenching, which can sharply reduce photodynamic efficiency.

### 3.5. Photophysical View of Zn-Porphyrin Aggregations

Aggregation is one of the main factors that reduce the PDT activity of porphyrins. Flat, hydrophobic macrocycles can easily form H- or J-aggregates through pi-pi interactions. In such aggregates, absorption bands shift, fluorescence decreases, the lifetime of excited states shortens, and the quantum yield of singlet oxygen drops. The high molecular extinction of a single molecule does not compensate for the loss of productive photochemistry if the majority of the photosensitizer is in a quenched aggregated state [17,28,48,51].

The central metal affects aggregation in several ways. First, it alters the planarity and electrostatic profile of the macrocycle. Second, it can provide axial coordination, which spatially prevents the tight packing of porphyrin planes. Third, the metal alters the affinity for proteins, lipids, and carriers. For Zn(II) porphyrins, axial coordination is particularly important since Zn(II) can interact with N-, O-, or S-donor groups in biomolecules or synthetic ligands. Properly selected axial or peripheral groups can reduce aggregation, increase solubility, and simultaneously direct the photosensitizer toward membranes or organelles [6,28,56].

The formulation often has the same significance as the chemical structure. Liposomes, micelles, polymeric nanoparticles, albumin complexes, porphyrin coordination polymers, and MOF systems can space out photosensitizer molecules, reduce self-quenching, and maintain oxygen access. This is particularly advantageous for Zn-porphyrins, as their photochemical efficiency is often high in the monomeric state but decreases significantly upon uncontrolled aggregation. Therefore, metallation and formulation should be considered as interrelated design levels rather than as independent stages of drug development [28,54,56,57].

A comparison of absorption, fluorescence, triplet photophysics, and aggregation allows us to generalize the specific role of Zn(II) in the photodynamic action of porphyrins.

### 3.6. The Photophysical Properties of Zn(II) in a Porphyrin PDT

Although a broad discussion of the influence of various central metals on the photophysics of porphyrins is presented above, the summary herein focuses on zinc (Zn(II)) complexes. This approach is methodologically justified, as zinc serves as a reference system in medicinal chemistry. It has a closed electron shell d10, which provides an optimal balance of properties: moderate fluorescence (useful in diagnostics) and high efficiency of intersystem crossing to the triplet state (crucial in photodynamic therapy, PDT).The photophysical profile of Zn(II) porphyrins can be described as a balance between three desirable properties: sufficiently strong absorption in the visible region, fluorescence suitable for tracking the photosensitizer, and the ability to form triplet states that effectively sensitize oxygen. Unlike many redox-active metals, Zn(II) typically does not introduce an aggressive metal-centered redox component, but rather modulates ligand-centered photochemistry. Unlike very heavy metals, it does not always maximize the ISC, but often provides a better compromise between photodiagnostics, phototoxicity, and biocompatibility [6,43,52].

From a mechanistic point of view, the central metal determines not a single isolated parameter, but a whole sequence of events: photon absorption, relaxation to lower singlet levels, competition between fluorescence and ISC, formation of the T1 state, energy or electron transfer, generation of ROS, and subsequent damage to biomolecules. For the rational design of PDT agents, this means that Zn-porphyrin cannot be evaluated merely by its absorption maximum or ^1^O_2_ quantum yield in a model solvent. An integrated characteristic is required, which includes monomeric stTE, spectral compatibility with the light source, triplet kinetics, stability, photobleaching, fluorescence suitability, localization within the cell, and behavior in the carrier [42,44,53].

Thus, photophysical properties take on biochemical significance when an excited photosensitizer begins to generate ROS.

## 4. The Central Metal and Mechanisms of Active Oxygen Species Generation in Photodynamic Therapy

The generation of ROS is a central biochemical process in photodynamic therapy (PDT). After absorbing a photon, the porphyrin photosensitizer transitions to an excited singlet state and then, provided there is efficient ISC, forms a long-lived triplet state. It is the triplet state that has a sufficiently long lifetime to interact with molecular oxygen, lipids, proteins, nucleotides, or low-molecular electron donors. In this context, the central metal of the porphyrin macrocycle cannot be regarded as a neutral structural insert. It alters the energy levels of the singlet and triplet states, the quantum yield of the ISC, redox potentials, the tendency to aggregate, binding to biomolecules, and the ratio between Type I and Type II mechanisms [7,8,16,58].

For Zn(II) porphyrins, this issue is of particular importance. Zn(II) is a d10 ion with a closed electron shell and does not typically play an active role in biological redox cycling. Therefore, unlike Fe-, Mn-, or Cu-porphyrins, Zn-porphyrin photosensitizers more often act as photochemical generators of ROS rather than as catalysts for repeated redox reactions. At the same time, Zn(II) can stabilize the planar or nearly planar geometry of the macrocycle, alter the symmetry of electronic transitions, enhance fluorescence, and support the formation of a triplet state suitable for oxygen sensitization [21,59,60,61]. Thus, the generation of ROS in Zn-porphyrin-PDT is determined not only by the presence of oxygen but also by the subtle interaction between the metal, the porphyrin ligand, peripheral substituents, and the cellular microenvironment [7,59,60].

After describing the triplet state, it is logical to move on to the Type II mechanism, which is the central pathway of photochemical cytotoxicity for many Zn-porphyrins.

### 4.1. Type II Mechanism: Energy Transfer and Singlet Oxygen

The Type II PDT mechanism is based on the transfer of energy from the triplet state of the photosensitizer to the ground triplet state of molecular oxygen. This results in the formation of singlet oxygen (^1^O_2_), an electronically excited form of O_2_ with high reactivity toward electron-rich biomolecules. Singlet oxygen rapidly reacts with the double bonds of unsaturated lipids, histidine, methionine, cysteine, tryptophan, and tyrosine residues in proteins, as well as with the guanine bases of nucleic acids [8,16,61,62]. Due to its short lifetime and small diffusion radius, its damaging effect is spatially limited: the greatest oxidation occurs wherever the photosensitizer is localized during irradiation [8,16,61].

Many Zn(II) porphyrins are effective type II photosensitizers because Zn(II) does not quench excited states as strongly as some paramagnetic or redox-active metals, but it is capable of altering the electronic structure of the macrocycle in such a way that the triplet state becomes sufficiently populated and energetically suitable for oxygen sensitization [35,59,60]. For example, water-soluble sulfonated or cationic Zn-porphyrins can exhibit a high capacity for ^1^O_2_ generation in an aqueous medium if their aggregation is limited and the porphyrin rings remain photochemically accessible [24,35,63]. Importantly, the mere presence of Zn(II) does not guarantee a high quantum yield of ^1^O_2_: hydrophobicity, charge, axial coordination, J- or H-aggregation, and incorporation into a nanomaterial can either enhance or suppress the Type II pathway [21,59,62].

The biological consequences of the Type II mechanism depend on subcellular localization. If Zn-porphyrin accumulates in the mitochondria, ^1^O_2_ oxidizes respiratory chain proteins, cardiolipin, and outer membrane proteins, which contributes to membrane depolarization, the release of cytochrome c, and the initiation of apoptosis. When localized in lysosomes, singlet oxygen damages membrane lipids and can cause lysosomal membrane permeabilization, leading to the release of cathepsins. When localized in the endoplasmic reticulum, it disrupts protein folding and calcium homeostasis and activates the response to misfolded proteins [22,33,64,65]. Thus, the Type II pathway is not merely a chemical energy-transfer reaction but a spatially organized biochemical process [35,58,63].

However, singlet oxygen does not exhaust all the possibilities of PDT; therefore, Type I radical processes must also be considered.

### 4.2. Type I Mechanism: Electron and Hydrogen Transfer

The Type I mechanism involves the transfer of an electron or a hydrogen atom between an excited photosensitizer and the substrate. The primary products may include a photosensitizer cation or anion radical, biomolecular radicals, the superoxide anion (O_2_•^−^), hydrogen peroxide (H_2_O_2_), the hydroxyl radical (•OH), and lipid radicals. Unlike the Type II pathway, the Type I mechanism does not necessarily begin with direct energy-driven oxygen sensitization; it may involve electron donors, NADH/NADPH-dependent systems, ascorbate, glutathione, membrane lipids, or protein residues [10,16,22,58,66,67,68,69,70,71].

The central metal influences the Type I mechanism through the porphyrin’s redox potentials, the stability of its radical forms, and the complex’s ability to accept or donate an electron. For Zn(II) porphyrins, the metal center is usually not the primary redox node, but metallation alters the electronic density of the macrocycle and can shift the redox properties of the entire complex [72,73,74,75]. Therefore, the Type I activity of Zn-porphyrin is often determined not by a direct Zn(II)/Zn(I) or Zn(III) cycle, but by the properties of the porphyrin π-system and peripheral substituents. Electron donor and electron acceptor groups, cationization, conjugation with biotin, or incorporation into nanoplatforms can alter the likelihood of superoxide and other ROS formation [24,38,50,60,64,68,69].

In Type I biological systems, this pathway plays a particularly important role in membrane damage. Radical processes can initiate chain peroxidation of polyunsaturated fatty acids, leading to the formation of lipid hydroperoxides, malondialdehyde, 4-hydroxynonenal, and other reactive aldehydes. Such products can damage not only the site of primary generation of ROS, but also distant proteins and signaling systems. Furthermore, superoxide can be converted to H_2_O_2_, which is less reactive but has a longer lifetime and is capable of diffusing over greater distances. In the presence of transition metals, H_2_O_2_ can participate in reactions leading to the formation of •OH, although Zn(II) itself does not catalyze the classical Fenton reaction [22,25,65,66,70].

Since both mechanisms can coexist, what matters in practice is not only their presence but also the correlation between them in a specific microenvironment.

### 4.3. The Balance Between Type I and Type II Mechanisms

In a real cell, PDT rarely occurs as a purely Type I or purely Type II process. Even a photosensitizer with a high quantum yield of ^1^O_2_ can, under certain conditions, trigger radical reactions, while a Type I-oriented complex can simultaneously sensitize singlet oxygen. The balance is determined by oxygen concentration, local viscosity, medium polarity, the presence of electron donors, photosensitizer concentration, light dose, irradiation intensity, aggregate state, and subcellular localization [8,16,58,66,67].

For type II Zn-porphyrins, the component often dominates under normoxic conditions, when O_2_ availability is sufficient and the photosensitizer is in a monomeric state. However, in dense tumor tissues, hydrophobic membranes, or aggregated nanostructures, the proportion of type I processes may increase. The mechanistic characterization of Zn-porphyrin-PDT should include not only a test for singlet oxygen but also the detection of superoxide, H_2_O_2_, lipid peroxidation, and photochemical radicals [21,25,35,59,66,67].

Aggregation is one of the key factors that alters the Type I/Type II balance. H-aggregates often reduce fluorescence and triplet yield, which suppresses ^1^O_2_. At the same time, dense aggregates or supramolecular structures can create local conditions conducive to electron transfer, especially if the system includes donor-acceptor fragments, semiconductor matrices, metal–organic frameworks, or other catalysts [22,24,69]. Thus, the central Zn(II) provides an important photophysical foundation, but the final profile of active oxygen species is the result of the overall molecular architecture [22,66,67].

The balance between the different types of reactions depends particularly on the nature of the central metal; therefore, the next step is to analyze its potential redox involvement.

### 4.4. Metal-Dependent Redox Chemistry

A comparison of Zn(II) porphyrins with Fe-, Mn-, and Cu-porphyrins demonstrates just how varied the role of the central metal can be. In many complexes, Zn(II), Mg(II), Al(III), or Sn(IV) primarily modulate the photophysics of the macrocycle, whereas Fe(III), Mn(III), and Cu(II) can directly participate in redox processes. Redox-active metal porphyrins are capable of catalyzing superoxide dismutation, the decomposition of peroxides, electron transfer, or the formation of highly oxidized metal-oxo species. Depending on their structure and environment, they can act as photosensitizers, pro-oxidant catalysts, or even as antioxidant-like modulators of ROS [7,50,60,70].

Therefore, Zn(II) porphyrin serves as a convenient model system for distinguishing “pure” porphyrin photochemistry from metal-catalyzed redox chemistry. If superoxide, H_2_O_2_, or lipid peroxidation are observed following Zn-porphyrin-PDT, these should first and foremost be attributed to the photoexcited π-system of the porphyrin, the microenvironment, and secondary cellular reactions, rather than to direct Zn redox cycling. This increases the predictability of the toxicological profile of Zn-porphyrins, but does not eliminate the need to monitor dark toxicity, photostability, metal impurities, and macrocycle degradation [24,59,60,63,70].

From a practical standpoint, the redox inertness of Zn(II) can be an advantage for therapeutic design. It reduces the risk of uncontrolled metal-dependent generation of ROS in the dark, makes the effect more dependent on light dose, and allows Zn-porphyrins to be used as theranostic agents, combining fluorescent imaging with photodynamic action. However, in a tumor environment, Zn-porphyrin is not isolated from other metals. Endogenous iron, copper, heme proteins, mitochondrial complexes, and a labile pool of metals can convert primary ROS into secondary reactive species. Therefore, the metal-dependent redox chemistry of PDT is a property not only of the photosensitizer, but also of the cellular microenvironment [50,60,64,65,70].

More information about the influence of the central metal is available in Table 1.

Understanding the type of ROS has direct implications for tumor hypoxia, where oxygen availability limits classical singlet-oxygen PDT.

### 4.5. Implications for Hypoxic Tumors

Hypoxia is one of the main limitations of PDT, since the classical Type II mechanism is directly dependent on molecular oxygen. Solid tumors often contain areas with low perfusion, high metabolic O_2_ consumption, acidic pH, and uneven distribution of the photosensitizer. During PDT, oxygen is further depleted, and vascular damage may exacerbate secondary hypoxia. Under these conditions, Zn-porphyrin, which acts primarily via ^1^O_2_, may demonstrate limited efficacy in deep or poorly vascularized tumor regions [50,58,67,68].

One approach to overcoming this problem involves shifting the ROS profile toward a combined Type I/Type II mechanism. This can be achieved by introducing electron donor or electron acceptor groups, cationic fragments, biotinylated or mitochondrially targeted substituents, as well as by incorporating Zn-porphyrin into nanoplatforms capable of delivering oxygen, catalyzing the decomposition of endogenous H_2_O_2_, or improving photosensitizer dispersion [22,50,68,69]. In such systems, Zn(II) retains the advantages of a stable photosensitizing center, while the peripheral or formulation environment adds the capacity for radical pathways [22,50,69].

Another approach involves controlling light dosimetry. Lower light intensity, fractionated irradiation, or pauses between light pulses can give the tissue time to reoxygenate and reduce rapid O_2_ depletion. This is particularly important for Zn-porphyrins with high Type II potential, as excessively intense irradiation can paradoxically reduce efficacy due to local oxygen depletion. Therefore, the optimization of PDT must take into account not only the molecule, but also the dynamics of oxygen in the tissue, the geometry of illumination, and the photochemical reaction rate [8,50].

In consequence, the central metal determines the primary photochemical pathway of the porphyrin photosensitizer, but it is not the sole regulator of ROS. Zn(II) porphyrins are often strong candidates for type II PDT due to their combination of redox inertness, favorable photophysics, and ability to generate singlet oxygen. However, in the tumor microenvironment, especially under hypoxic conditions, their efficacy depends on whether the system can support additional Type I reactions, prevent aggregation, achieve proper subcellular localization, and maintain controlled phototoxicity. These factors make an understanding of metal-dependent active oxygen species generation the foundation for the rational design of the next generation of Zn-porphyrin PDT agents [8,50,60,66,76,77,78,79,80,81,82,83,84,85].

Once the mechanisms by which ROS are formed have been established, it is necessary to determine exactly where these particles are formed within the cell, since their action is short-range.

### 4.6. Effect of the Central Metal on Subcellular Localization

The subcellular localization of the photosensitizer is one of the key factors that transforms a general photochemical reaction into a specific biological response. ROS, particularly singlet oxygen, have a short lifetime and a small diffusion radius within the cell; therefore, the primary targets of PDT are mostly located close to the site of photosensitizer accumulation [86,87,88]. If the porphyrin is located in the mitochondrial membrane, early events include depolarization, the release of cytochrome c and the initiation of intrinsic apoptosis. If it accumulates in lysosomes, lysosomal membrane permeabilization, the release of cathepsins and links to the autophagic or necrotic response predominate. If the photosensitizer is associated with the endoplasmic reticulum, protein oxidation, calcium dysregulation and the response to misfolded proteins become important. Therefore, localization is not merely a pharmacokinetic detail; it determines which organelles will be primarily damaged, what type of cell death is triggered, and to what extent the therapeutic response will be selective [58,64,79,80,81,82]. For Zn(II) porphyrins, localization is determined not only by peripheral substituents but also by the metallation of the macrocycle itself. The Zn(II) ion alters planarity, dipole moment, axial coordination capacity, aggregation, protein affinity, and interaction with membranes. Since Zn(II) is a closed-shell d10 center and does not typically undergo redox cycling under biological conditions, its contribution to localization is predominantly structural and coordinative, rather than catalytic. Consequently, Zn-porphyrin can act as a fluorescent, photochemically active and, at the same time, coordination-sensitive probe capable of moving between protein, membrane and nanoformulation microenvironments [6,12,24,80,83,84,85,86].

Analysis of localization begins with cellular uptake, as the route of entry of the photosensitizer often determines its subsequent organellar distribution.

## 5. Cellular Uptake: Endocytosis, Passive Diffusion, Serum Proteins and Lipoproteins

The entry of porphyrin photosensitizers into the cell depends on their hydrophobicity, charge, molecular size, degree of aggregation and delivery form. Hydrophobic or amphiphilic porphyrins can associate with the plasma membrane, serum albumin, low- and high-density lipoproteins, as well as with membrane domains, after which they enter the cell via passive diffusion or endocytosis [86,87,88]. More hydrophilic, charged or nanocapsulated systems more frequently utilize the clathrin-dependent, caveolin-dependent or micropinocytosis pathways. This is important for PDT because endocytosis primarily directs molecules to the endosomal-lysosomal compartment, whereas membrane diffusion and lipoprotein transport may increase the likelihood of mitochondrial or membrane accumulation [79,81,82].

Zn(II) porphyrins often exhibit high affinity for proteins and lipid microenvironments. The central Zn(II) may coordinate nitrogen- or oxygen-containing donor groups, including imidazole residues of histidine, carboxylates or heterocyclic ligands. Such coordination does not necessarily constitute a stable covalent bond, but it can alter the orientation of the photosensitizer near a protein surface or within a membrane. In serum, this affects transport, availability to tumor tissue and dark toxicity; within the cell, it affects distribution between membranes, the cytosol and organelles. It is therefore insufficient to evaluate Zn-porphyrin merely as a small molecule in buffer: within a biological system, it exists as an ensemble of complexes with proteins, lipids and nanostructures [6,83,86].

Having described the uptake pathways, it is important to move on to the molecular properties that determine the selection of membrane and organelle targets.

### 5.1. Charge and Amphiphilicity as Determinants of Organelle Targeting

Peripheral charge is one of the strongest predictors of the subcellular localisation of porphyrins. Cationic porphyrins interact more effectively with negatively charged membranes, bacterial envelopes, mitochondria and nucleic acids. Anionic derivatives are more likely to remain in the extracellular environment or enter the cell via endocytosis. Neutral and amphiphilic structures may pass more easily through lipid bilayers, but high hydrophobicity increases aggregation and non-specific binding to membranes [51,87,88]. The optimal molecule is not usually the most highly charged or the most lipophilic; it possesses a balanced amphiphilicity that allows it to avoid coarse aggregation, maintain a monomeric state and reach the target organelle [12,81,86].

The introduction of Zn(II) into the porphyrin core alters this balance indirectly. Metallation may reduce the internal proton mobility of the free-base porphyrin, increase symmetry and influence the distribution of electron density. Furthermore, the Zn(II) center opens up the possibility of axial functionalization. Through axial ligands, fragments can be attached to Zn-porphyrin that increase water solubility, confer mitochondrial targeting, reduce aggregation or introduce receptor recognition. Thus, the central metal acts as a hub in the molecular architecture: the peripheral substituents determine the overall amphiphilicity, whilst axial coordination provides an additional level of control over localization [80,83,87].

Among organellar targets, mitochondria are of particular importance, as their damage directly links photochemistry to regulated cell death.

### 5.2. Mitochondrial Localisation: Membrane Potential, Cardiolipin and Apoptosis

Mitochondria are a particularly sensitive target for PDT, as they combine a high content of membrane lipids, localized oxygen, redox-active protein complexes and a central role in the regulation of apoptosis. Cationic and lipophilic photosensitizers can accumulate in mitochondria due to the negative membrane potential of the inner membrane. Upon illumination, the oxidation of cardiolipin, respiratory chain proteins and membrane components leads to depolarization, disruption of ATP synthesis, the opening of the transitional pore, the release of cytochrome c and the activation of caspases [84,89,90]. In the case of moderate damage, the cell may initiate mitophagy as a protective process; in the case of severe or rapid damage, apoptosis or necrotic death predominates [79,88,91].

Zn-porphyrins can be directed toward mitochondria by the introduction of cationic groups, triphenylphosphonium moieties, pyridinium substituents, or amphiphilic chains. Their advantage lies in the fact that Zn(II) supports fluorescent monitoring of localization and the ability to generate singlet oxygen, but does not introduce a strong intrinsic metal redox cycle. However, excessive cationic character may increase dark toxicity and non-specific binding to membranes. Therefore, for mitochondria-targeted Zn-porphyrins, it is not only phototoxicity following illumination that is important, but also the light toxicity/dark toxicity ratio, the clearance rate, and the ability to maintain a monomeric state in the mitochondrial membrane [12,87,91].

Alongside mitochondria, it is important to consider lysosomes, as their membrane permeabilization can trigger secondary damage cascades.

### 5.3. Lysosomal Localization: Membrane Permeabilization, Cathepsins and Secondary Mitochondrial Damage

Lysosomes often serve as the primary site of accumulation for photosensitizers that enter the cell via endocytosis or possess lysosomotropic amine groups. The acidic environment, high concentration of hydrolases and abundance of membrane phospholipids make lysosomes vulnerable to local photogeneration of ROS. PDT-induced lysosomal membrane permeabilization leads to the release of cathepsins into the cytosol, the cleavage of Bid protein, disruption of the autophagic flux, activation of inflammatory signals and secondary damage to mitochondria [90,91,92,93,94,95,96,97,98,99,100,101]. Lysosomal PDT may be particularly effective when cells have a high endocytic flux or are dependent on autophagy for survival [82,88,92].

For Zn-porphyrins, lysosomal localization can be both desirable and limiting. On the one hand, endocytosis of nanoformulations or protein complexes often naturally directs the photosensitizer to lysosomes, where light activation causes rapid organelle damage. On the other hand, lysosomal ‘capture’ may hinder access to mitochondria, the endoplasmic reticulum (ER) or the plasma membrane, if these specific targets are required for a particular therapeutic scenario. Furthermore, the acidic environment of lysosomes and protein-lipid complexes may alter the spectral properties and aggregation state of Zn-porphyrins. Therefore, studies of localization should include not only colocalization with LysoTracker, but also an assessment of phototoxicity, lysosomal stability, the autophagic flux and the link to mitochondrial consequences [88,92,93].

Lysosomal and mitochondrial mechanisms are complemented by damage to the cell’s secretory system, particularly the endoplasmic reticulum and the Golgi apparatus.

### 5.4. The Endoplasmic Reticulum and the Golgi Apparatus: ER Stress, Calcium and Protein Homeostasis

The endoplasmic reticulum is the site of synthesis of secretory and membrane proteins and regulates protein folding, calcium storage and lipid metabolism. ER-targeted FDT can oxidize chaperone system proteins, disrupt disulfide exchange, alter calcium fluxes and trigger a response to misfolded proteins via PERK, IRE1/ERN1 and ATF6 [82,93]. If the damage is moderate, ER stress may promote survival through temporary inhibition of translation, upregulation of chaperones and degradation of misfolded proteins. If the damage exceeds the cell’s adaptive capacity, the UPR shifts to a pro-apoptotic mode via CHOP, JNK signaling, calcium transmission to the mitochondria and caspase activation [87,91,93].

The localization of Zn-porphyrins in the ER may be due to their amphiphilic nature, binding to membranes, protein complexes or specific targeting moieties. The axial coordination capacity of Zn(II) could theoretically facilitate interactions with ER proteins containing donor groups, although such interactions are typically weak and context-dependent within the cell. The Golgi apparatus, like the ER, has a branched membrane system and is involved in protein modification and sorting; damage to it can disrupt secretion, glycosylation and membrane trafficking. For experimental interpretation, it is important to distinguish between true ER/Golgi targeting and the temporary presence of the photosensitizer in the endomembrane transport pathway following endocytosis [51,86,93].

In addition to intracellular organelles, the photosensitizer may act on the plasma membrane and vascular structures, thereby altering the scale of the response from the cellular to the tissue level.

### 5.5. The Plasma Membrane, Vascular Targeting and Surface Damage

The plasma membrane is the primary target for photosensitizers that remain on the cell surface or are strongly associated with the lipid bilayer. The oxidation of unsaturated fatty acids, cholesterol, receptors, transporters and cytoskeletal-membrane complexes can rapidly disrupt membrane permeability, ion balance, adhesion and the mechanical integrity of the cell [72,91]. At high doses of light or photosensitizer, membrane PDT often leads to necrosis or necrosis-like cell death due to the loss of the barrier function. In tumor tissue, vascular targeting is of particular significance: photodynamic damage to the endothelium can cause vasoconstriction, thrombosis, increased vascular permeability and secondary tumor hypoxia [72,79,81].

Zn-porphyrin derivatives with long alkyl chains, amphiphilic groups or protein-lipid carriers may exhibit significant membrane affinity. This is beneficial for antimicrobial PDT, where superficial damage to cell membranes is often therapeutically desirable; however, for antitumor PDT, excessive non-specific membrane binding may increase phototoxicity to normal cells. Consequently, membrane localization must be assessed alongside delivery selectivity, the light field and tissue pharmacokinetics [6,24,28].

Having considered the individual compartments, we should return to Zn(II) as a factor that, through planarity, coordination and aggregation, influences the distribution of the molecule within the cell.

### 5.6. Zinc-Dependent Localization Effects: Planarity, Axial Coordination, Lipophilicity and Aggregation

The central Zn(II) rarely determines organelle localization on its own, but it significantly alters the probability of different distribution pathways. A planar Zn-porphyrin macrocycle readily interacts with the hydrophobic domains of membranes and proteins; peripheral substituents govern solubility and charge; axial ligands may reduce aggregation or impart additional directionality. If Zn-porphyrin aggregates in an aqueous medium, its cellular uptake may increase via endocytosis, but photodynamic efficiency is often reduced due to self-quenching. If the formulation maintains a monomeric or controlled-separation state, fluorescence, singlet oxygen generation and localization reproducibility increase [12,24,28,83,84,87].

Thus, the localization of Zn-porphyrins results from a multi-level structure: the central metal provides the coordination and photophysical platform; the peripheral groups determine the charge, amphiphilicity and bio-recognition; the carrier controls aggregation, delivery and the endocytic pathway; the cellular microenvironment modifies protein binding, pH-dependent behavior and organelle distribution. The best Zn-porphyrin photosensitizer for PDT is not necessarily the one that produces the most ^1^O_2_ in solution. The optimal system is one in which the photogeneration of ROS spatially coincides with a biologically critical target: the mitochondria, lysosomes, endoplasmic reticulum, cell membrane, endothelium or tumor stromal component. It is precisely this spatial-chemical correspondence that transforms metal porphyrins PDT from non-specific oxidative stress into a controlled mechanism of cellular damage [28,72,79,91].

**Figure 4:**
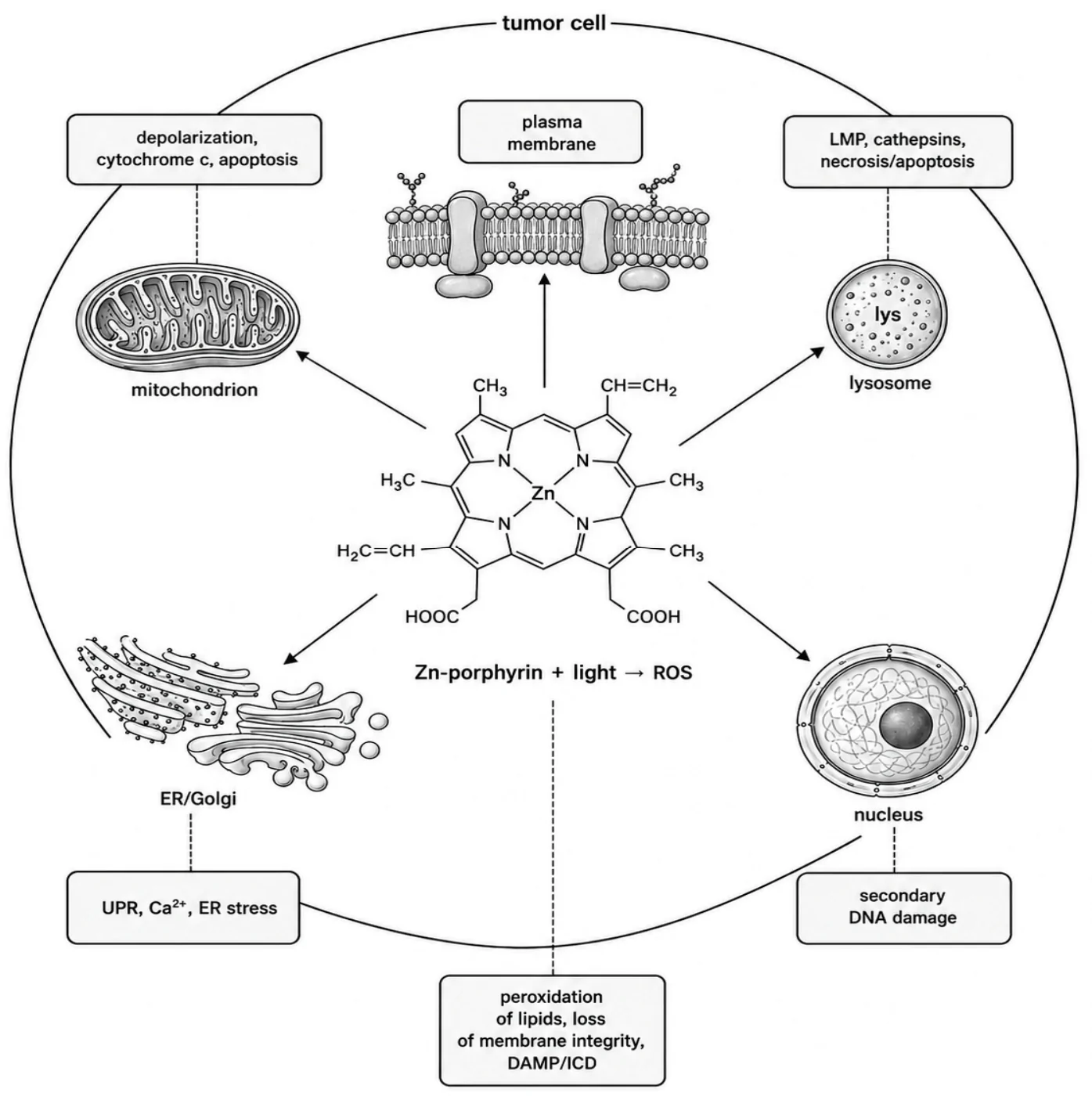
Subcellular mechanisms of Zn-porphyrin PDT.

Table 2 summarizes the subcellular localization of Zn-porphyrin photosensitizers and expected mechanistic consequences [28,79,80]. Because most PDT-derived reactive species act over short distances, subcellular localization determines the primary oxidized targets and the subsequent mode of cellular injury, as summarized in Figure 4.

Once the site of ROS formation has been identified, the next level of analysis involves determining which biomolecules are damaged first and how this translates into a cellular response.

## 6. Biochemical Mechanisms of Cell Damage Induced by Zn-Porphyrin Photodynamic Therapy

Zn-porphyrin photodynamic therapy (PDT) exerts its cytotoxic effect through a cascade of spatially confined oxidative reactions. Zinc-porphyrin absorbs light to reach a singlet excited state, then rapidly undergoes ISC to form a long-lived triplet state. This triplet state plays a vital role in light-driven processes like solar energy conversion and artificial photosynthesis. Since Zn(II) is a d10 ion and does not normally participate in intensive biological redox cycling, the primary toxicity of such photosensitizers is determined predominantly not by direct metal-catalyzed chemistry, but by the photophysics of the porphyrin macrocycle, subcellular localization, aggregation state, oxygen availability and light dose. Therefore, the biochemical profile of the damage depends on the exact location of the molecule during irradiation: in membranes, mitochondria, the endoplasmic reticulum, lysosomes or near the nucleus [72,78,102].

Proteins are among the most sensitive targets of photodynamic oxidation; therefore, it is advisable to begin the analysis of biochemical damage with them.

### 6.1. Protein Oxidation

Proteins are among the most important primary targets of Zn-porphyrin PDT. Singlet oxygen has a short lifetime and a limited diffusion range within the cell; consequently, it predominantly modifies those proteins located in close proximity to the photosensitizer. The most sensitive amino acid residues are cysteine, methionine, histidine, tryptophan and tyrosine. The oxidation of cysteine alters the state of thiols and disulfide bonds; it can inactivate enzyme active sites, disrupt redox-sensing domains and alter protein–protein interactions. Methionine residues are converted into sulfoxides; this process is sometimes reversible, but at high light doses it leads to permanent damage. The oxidation of histidine, tryptophan and tyrosine affects the spatial structure of proteins, receptor stability, transporter function, enzyme activity and cytoskeletal organization [72,78,106,107,108].

The topology of the photosensitizer determines which proteins are damaged first. Mitochondrially localized Zn-porphyrins predominantly affect respiratory chain proteins, outer mitochondrial membrane proteins, and membrane permeability regulators. Lysosomal localization directs oxidation toward lysosomal membrane proteins, cathepsins and proteins of the endocytic pathway. When localized in the endoplasmic reticulum, chaperones, folding proteins, disulfide bond-forming proteins, and Ca^2+^-dependent regulatory complexes become critical. Thus, the same overall production of ROS can lead to different response phenotypes: adaptive stress, apoptosis, the autophagic response, necrosis or immunogenic cell death [96,108,109].

Following the discussion of protein targets, it is necessary to consider membrane lipids, as their peroxidation often determines membrane permeability and the type of subsequent cell death.

### 6.2. Lipid Peroxidation

The second fundamental level of photodynamic damage is lipid peroxidation. Unsaturated phospholipids in biological membranes readily undergo oxidation reactions, particularly if the Zn-porphyrin possesses lipophilic or cationic peripheral groups and accumulates in the membrane environment. Singlet oxygen can directly oxidize the double bonds of unsaturated fatty acids to form lipid hydroperoxides. Type I radical processes further trigger chain peroxidation, in which a single primary radical can damage many lipid molecules. As a result, membrane fluidity is reduced, the function of ion channels is altered, the activity of membrane enzymes is disrupted, and the barrier function of plasma and organelle membranes is impaired [74,99,110].

The products of lipid peroxidation are not limited to local membrane toxicity. Reactive aldehydes, in particular 4-hydroxy-nonenal and malondialdehyde, can covalently modify proteins, nucleic acids and signaling molecules. This transforms the local photochemical process into a broader cellular stress program. In mitochondria, the oxidation of cardiolipin is of particular significance, as it stabilizes the respiratory chain complexes and interacts with cytochrome c. Peroxidation of cardiolipin promotes the detachment of cytochrome c from the inner membrane, the disorganization of cristae, a reduction in membrane potential and the activation of the apoptotic cascade. In this sense, Zn-porphyrin PDT may combine direct membrane damage with the subsequent activation of organelle-mediated death mechanisms [37,99,110,111].

Lipid and protein oxidation is particularly critical in mitochondria, where it rapidly leads to energy and apoptotic dysfunction.

### 6.3. Mitochondrial Dysfunction

Mitochondria are the central biochemical hub of PDT, as they integrate energy metabolism, control of ROS, Ca^2+^ regulation and the initiation of apoptosis. If Zn-porphyrin accumulates in or near the mitochondria, the photooxidation of respiratory chain proteins and membrane lipids rapidly reduces the efficiency of oxidative phosphorylation. Disruption of mitochondrial complexes I–IV enhances electron leakage to oxygen, leading to the formation of secondary mitochondrial ROS. These secondary ROS are no longer a direct product of the photosensitizer, but they amplify the initial damage and exacerbate the loss of cellular homeostasis [37,94,96,102,106,108].

The key consequences of mitochondrial damage include depolarization of the inner membrane, reduced ATP synthesis, the opening of permeability channels, an altered balance of the BCL-2 family, and the release of cytochrome c, Smac/DIABLO and other pro-apoptotic factors. In the case of moderate damage, the cell can activate mitophagy, antioxidant systems and metabolic reprogramming. In the case of severe damage, the loss of ATP and massive membrane disruption shift the response toward caspase-dependent or necrotic cell death [37,96,108].

Alongside the mitochondria, the endoplasmic reticulum is a key site of the response, where oxidative damage disrupts protein folding and calcium homeostasis.

### 6.4. ER Stress and the Response to Misfolded Proteins

The endoplasmic reticulum is particularly sensitive to Zn-porphyrin PDT when the photosensitizer is localized in the ER, the Golgi apparatus or membranes functionally associated with the ER. Oxidation of folding proteins, disruption of the disulfide balance and changes in Ca^2+^ homeostasis lead to the accumulation of misfolded proteins. In response, the unfolded protein response (UPR) is activated, comprising three main sensory pathways: PERK, IRE1/ERN1 and ATF6. PERK phosphorylates eIF2α and temporarily inhibits overall translation, thereby reducing the load on the ER. IRE1/ERN1 mediates the splicing of XBP1 and may activate JNK-dependent signaling. Following processing in the Golgi apparatus, ATF6 activates the transcription of genes encoding chaperones and components of ER-associated degradation [97,106,109,112].

In its early phase, the UPR plays an adaptive role: it reduces the production of new proteins, enhances the chaperone response and activates the degradation of misfolded proteins. However, in the event of excessive photodynamic damage, this program shifts into a pro-apoptotic mode. Importantly, ER stress is closely linked to mitochondria via Ca^2+^ signaling. Photodamage to the ER can cause Ca^2+^ to leak into the cytosol and overload the mitochondria, which enhances depolarization, the amplification of ROS, and the release of cytochrome c. Consequently, Zn-porphyrin PDT often does not damage a single organelle in isolation, but rather forms an ER-mitochondrial stress axis, in which the initial localization of the photosensitizer determines the direction of the subsequent cellular response [97,109,112].

ER stress and the accumulation of damaged proteins logically lead to an analysis of the cellular clearance systems—the proteasome and autophagy.

### 6.5. The Proteasome and Autophagy

The proteasome system is the first to respond to the accumulation of oxidized proteins. Moderately damaged proteins can be recognized by the 20S or 26S proteasome and removed, which temporarily protects the cell from proteotoxicity. However, intense PDT simultaneously increases the number of oxidized substrates and may damage the proteasome components themselves. Under such conditions, the cell loses its ability to maintain proteostasis; protein aggregates accumulate, and chaperones, the UPR and compensatory autophagy are activated. This explains why pharmacological inhibition of the proteasome or chaperone systems may enhance phototoxicity: it limits the tumor cell’s ability to restore protein homeostasis following photooxidation [93,97,113,114].

Autophagy following Zn-porphyrin PDT is twofold in nature. At moderate stress levels, it performs a cytoprotective function: it removes oxidized protein aggregates, damaged mitochondria, ER fragments and partially damaged lysosomal structures. Such a response may reduce the efficacy of PDT and promote the survival of tumor cells. At the same time, in the event of massive damage or blockage of the lysosomal stage, the autophagic flux becomes ineffective, autophagosomes accumulate, and the cell transitions to death-associated autophagy or mixed forms of cell death. Therefore, it is insufficient to interpret autophagy solely based on LC3-II levels; it is necessary to assess the autophagic flux, p62/SQSTM1, lysosomal activity and long-term clonogenic survival [93,96,97,113,114].

Although DNA is often not the primary target of porphyrin-mediated PDT, damage to it may occur secondarily and influence the long-term cellular response.

### 6.6. DNA Damage

DNA in Zn-porphyrin PDT is generally not the primary target unless the photosensitizer accumulates in the nucleus or has a specific DNA-affinity structure. The reason lies in the short diffusion distance of singlet oxygen: ROS predominantly damage the structures near which they are generated. Therefore, for most porphyrin photosensitizers without nuclear targeting, the primary targets are membranes, proteins, mitochondria, the ER, and lysosomes, while DNA damage is secondary in nature [72,102,107].

Secondary DNA damage may arise through diffuse ROS, products of lipid peroxidation, and disruption of mitochondrial metabolism. Possible consequences include the formation of 8-oxoguanine, single- and double-strand breaks, activation of ATM/ATR and p53-dependent responses, cell cycle arrest, and initiation of repair. If the damage is minor, the cell may restore genomic integrity. If it is combined with ATP deficiency, mitochondrial dysfunction, and ER stress, DNA damage becomes part of an integrated cell death program. In the context of Zn-porphyrins, this highlights the importance of not only overall phototoxicity but also precise control of subcellular localization [96,101,107,110].

The targets discussed should be integrated into a coherent framework in which proteins, lipids, organelles, and genetic material form an interconnected damage profile.

### 6.7. Summary of the Biochemical Response

The overall biochemical picture of Zn-porphyrin PDT is shaped by the interplay of three parameters: the photochemical efficiency of the Zn-porphyrin, its microlocalization, and the adaptive capacity of the cell. When ROS are generated near mitochondria, depolarization, cytochrome c release, and apoptosis predominate. When the ER is primarily damaged, UPR, Ca^2+^ imbalance, and ER–mitochondria crosstalk become the leading processes. When membranes are the main target, lipid peroxidation, loss of barrier function, ferroptosis-like signaling, and necrotic components come to the fore. A Zn-porphyrin photosensitizer should therefore not be evaluated solely by its singlet oxygen quantum yield: the therapeutic effect is determined by the “structure–localization–biochemical response” relationship [72,78,96,102,108,109].

Thus, Zn(II) in the porphyrin core provides a favorable combination of photosensitizing activity and relative redox biocompatibility, while the ultimate phenotype of cellular damage is defined by peripheral substituents, formulation, aggregation, oxygen availability, light dose, and organellar targeting. The rational design of Zn-porphyrins should aim not merely at maximizing ROS generation, but at controlled damage to specific biochemical nodes within the tumor cell [72,78,80,111,115].

Biochemical damage acquires therapeutic significance when it shifts the cell toward a particular pathway of death or adaptive response.

## 7. Cell Death Pathways Activated by Metal Porphyrin Photodynamic Therapy

Photodynamic therapy (PDT) is not a one-size-fits-all approach to cytotoxicity. After photoactivation of a porphyrin or metal porphyrin photosensitizer, the cell undergoes a spatially limited oxidative shock, and its subsequent fate is determined by the localization of the photosensitizer, the light dose, oxygen concentration, incubation time, the cell’s redox state, the activity of antioxidant systems, and the cell’s ability to repair damage [12,13,27,116]. This is particularly important for Zn(II) porphyrins, since zinc typically does not act as a redox-active center similar to Fe, Mn, or Cu, and the primary biological effect results from the photoinduced generation of singlet oxygen and ROS in specific organelles [32,117,118]. Therefore, the same class of Zn-porphyrin photosensitizers can trigger apoptosis, necrosis, autophagy-associated cell death, ferroptosis-like events, or immunogenic cell death, depending on the biophysical context of PDT [27,93,116].

Among the possible consequences of moderate photodynamic damage, apoptosis is the most organized and therefore requires priority consideration.

### 7.1. Apoptosis

Apoptosis is one of the most common consequences of moderate photodynamic damage when the cell still retains the energy resources necessary to carry out a regulated program of cell death [13,27,119]. For porphyrin-based PDT, the most characteristic pathway is the intrinsic, or mitochondrial, pathway of apoptosis. If the photosensitizer is localized in or near the mitochondria, singlet oxygen and radical products rapidly damage cardiolipin, respiratory chain proteins, membrane lipids, and regulators of the BCL-2 family [102,119]. This promotes the activation of BAX/BAK, mitochondrial outer membrane permeabilization, the release of cytochrome c, the formation of the apoptosome, and caspase activation [119,120].

For Zn-porphyrins, apoptosis is often associated not with direct metal-redox chemistry, but with the high local efficiency of oxygen photosensitization. If a molecule has cationic, amphiphilic, or lipophilic substituents, it can accumulate in mitochondria due to the membrane potential or binding to membrane structures; in such cases, the Zn(II) center indirectly enhances apoptosis through favorable photophysical properties rather than through redox cycling itself [6,93,117]. At lower light doses, apoptosis may be relatively pure and caspase-dependent, whereas at higher doses it is often combined with necrosis or necroptosis [13,27,121].

Molecular markers of apoptosis following PDT include a decrease in mitochondrial membrane potential, activation of caspases-9 and -3, PARP cleavage, translocation of phosphatidylserine to the outer leaflet of the plasma membrane, and DNA fragmentation [79,119,120]. However, these markers must be interpreted with caution: PDT can simultaneously damage lysosomes, the endoplasmic reticulum (ER), and the plasma membrane; therefore, the Annexin V/PI profile or caspase activity does not always reflect a single mechanism of cell death [27,116,121].

If the severity of the damage exceeds the cell’s ability to sustain an energy-dependent apoptotic program, the response shifts toward necrotic forms.

### 7.2. Necrosis and Regulated Necrosis

Necrosis predominates when photodynamic stress is too intense for an orderly apoptotic program to occur. High concentrations of the photosensitizer, excessive light doses, plasma membrane localization, or rapid ATP depletion lead to a loss of ionic homeostasis, cell swelling, membrane rupture, and the release of intracellular contents [13,27,37]. In tissue, this can have dual implications: on the one hand, necrosis reduces the controllability of cytotoxicity; on the other hand, it promotes the release of DAMPs and enhances inflammation, which may support the antitumor immune response [119,120,121,122,123,124,125].

A distinct variant is regulated necrosis, specifically necroptosis. Unlike passive necrosis, necroptosis is associated with the activity of RIPK1, RIPK3, and MLKL and culminates in the formation of pores in the plasma membrane [121,123]. Recent reviews indicate that PDT can induce necroptosis in various tumor cells, and this process often coexists with apoptosis, autophagy, or other forms of cell death [27,121]. This is important for metal porphyrin PDT because the choice of central metal, charge, and localization of the photosensitizer can alter the balance between the caspase-dependent and necroptotic responses [116,117,121].

To experimentally distinguish between necrosis and necroptosis, it is advisable to combine morphological analysis with measurements of LDH, PI permeability, ATP levels, and MLKL phosphorylation, as well as with pharmacological inhibition of RIPK1/RIPK3/MLKL [121,123]. If, following caspase inhibition, cells do not survive but proceed to MLKL-dependent membrane disruption, this indicates a switch between apoptosis and necroptosis [121].

Autophagy lies between adaptation and cell death; following PDT, it can be either protective or damaging.

### 7.3. Autophagy-Associated Cell Death

Autophagy following PDT has a dual role. At moderate levels, it is an adaptive response that removes oxidized proteins, damaged mitochondria, and membrane fragments, temporarily increasing cell survival [93,124]. However, under severe or prolonged stress, the autophagic response can shift to death-associated autophagy, especially if organelle damage exceeds the cell’s repair capacity [64,93,124]. Therefore, inhibiting autophagy enhances PDT in some models but reduces phototoxicity in others, provided that autophagy was part of the lethal mechanism [64,124].

For Zn-porphyrin PDT, autophagy often occurs in response to mitochondrial, ER or lysosomal damage [93,117,118]. Oxidation of mitochondrial membranes can activate mitophagy, ER stress can activate ER-phagy or a general UPR-dependent autophagic flux, and lysosomal photodestruction disrupts the final stage of autophagy, since the degradation of autophagosomes depends on functional lysosomes [93,124].

In such a situation, the accumulation of LC3-II or p62/SQSTM1 does not always indicate enhanced autophagy; it may reflect a block in the autophagic pathway [93].

From a methodological standpoint, autophagy-associated cell death cannot be determined solely by an increase in LC3-II. Analysis of p62, LC3-staining microscopy, the use of inhibitors of early and late stages of autophagy, assessment of viability following genetic or pharmacological inhibition of ATG proteins, and differentiation between cytoprotective and cytotoxic autophagy are required [64,93,124].

Since PDT actively damages membrane lipids, ferroptosis-like processes associated with lipid peroxidation require special attention.

### 7.4. Ferroptosis-like Mechanisms

Ferroptosis is an iron-dependent form of regulated cell death based on the accumulation of toxic products of polyunsaturated lipid peroxidation, glutathione depletion, insufficient GPX4 activity, and disruption of lipid redox homeostasis [125,126,127]. PDT naturally overlaps with ferroptosis, as the photoactivated photosensitizer generates ROS that trigger or enhance the peroxidation of membrane lipids [27,125,126]. In tumors resistant to apoptosis, such an interaction may be therapeutically beneficial, especially when PDT is combined with ferroptosis inducers or inhibitors of antioxidant defense [125,126,127].

For Zn-porphyrins, it is more accurate to speak of ferroptosis-like or ferroptosis-associated responses, unless a dependence on labile iron, GPX4, and the specific inhibition of ferroptosis by ferostatin-1, liproxstatin-1, or iron chelators has been demonstrated [117,125,126,127]. Zn-porphyrin-mediated PDT may indirectly promote ferroptosis through lipid peroxidation, mitochondrial damage, disruption of the glutathione pool, and impairment of the SLC7A11-GSH-GPX4 system [122,126].

To demonstrate ferroptosis in metal porphyrins PDT models, it is necessary to measure C11-BODIPY oxidation, 4-HNE/MDA and GSH/GSSG levels, and the expression or activity of GPX4, ACSL4, and SLC7A11, as well as to conduct rescue experiments using ferroptosis inhibitors [125,126]. Without such a data set, lipid peroxidation following PDT remains important but insufficient evidence of true ferroptosis [125].

In addition to direct cell destruction, PDT can transform localized damage into an immune signal, making immunogenic cell death particularly important.

### 7.5. Immunogenic Cell Death

Immunogenic cell death (ICD) is a particularly valuable outcome of PDT, as localized photodynamic damage can transform the tumor into a source of antigens and danger signals [116,122,128]. Classic markers of ICD include the exposure of calreticulin on the cell surface, the release of ATP, HMGB1, HSP70/HSP90, oxidized lipids, and other DAMPs, which activate dendritic cells, macrophages, and neutrophils [116,122,128]. Dendritic cells absorb antigens from dead tumor cells, mature, migrate to lymph nodes, and present antigens to CD8+ T cells, thereby eliciting a systemic antitumor response [122,128].

PDT is one of the most promising physicochemical approaches to inducing ICD, as it simultaneously generates local oxidative stress, damages tumor cells, affects blood vessels, and can alter the inflammatory microenvironment [116,122,128]. For Zn-porphyrins, ICD does not depend on the toxicity of Zn(II), but rather on whether the photosensitizer is sufficiently effective at generating ROS in the ER, mitochondria, or plasma membrane.

In a translational context, ICD explains why PDT can potentially be combined with immune checkpoint inhibitors, vaccination approaches, nanoformulations, and adjuvant strategies [116,122,128]. However, to draw a valid conclusion about ICD, not only in vitro DAMP markers but also functional data are required: dendritic cell maturation, T-cell activation, protective antitumor vaccination, or reduced growth of a re-implanted tumor in immunocompetent models [116,122,128].

After examining individual pathways of cell death, it is necessary to summarize how the central metal indirectly modulates the cellular response through photophysics and localization.

### 7.6. Summary: Metal as a Regulator of the Type of Cell Death

The type of cell death following metal porphyrin-mediated PDT is an integral result of photochemistry, localization, and the biological state of the cell. The metal at the center of the porphyrin alters light absorption, ISC, the triplet state, the profile of ROS, aggregation, and interactions with biomolecules, and consequently—the distribution among apoptosis, necrosis, autophagy, ferroptosis-like processes, and ICD [27,32,116,117,118].

Thus, Zn-porphyrin PDT is a good example of how a “non-redox-active” metal can have a significant impact on the biological outcome [27,93,116,122,125,128]. Therefore, the future design of Zn-porphyrin photosensitizers should be aimed not only at high phototoxicity but also at the controlled programming of the desired type of cell death. For anticancer therapy, the most promising approach is not the isolated induction of a single mechanism, but rather a rational combination of a local cytotoxic effect with immunogenic, metabolic, and microenvironmental responses [116,122,128].

To more accurately assess the contribution of Zn(II), its effects should be compared with the behavior of metal-free porphyrins on the same macrocyclic platform.

## 8. Metal-Free Porphyrins Compared to Zn-Porphyrins

Comparing metal-free porphyrins with Zn(II) porphyrins is essential for understanding how central coordination alters the photodynamic mechanism. In both cases, the active light-absorbing platform is a tetrapyrrole macrocycle, but the presence or absence of a central cation affects the molecular symmetry, spectral structure, fluorescence, triplet state formation, singlet oxygen generation, aggregation, photostability, biodistribution, and therapeutic window in different ways [6,71,129,130]. Metal-free porphyrins retain two internal N-H protons and have lower effective symmetry, whereas Zn(II) forms a coordination complex with the four nitrogen atoms of the porphyrin core. This structural change, which may seem minor at first glance, can alter not only the spectroscopic parameters but also the practical profile of the photosensitizer for PDT [121,129].

The comparison should begin with photophysics, as it is precisely photophysics that responds most rapidly to the presence or absence of the central metal.

### 8.1. Photophysical Comparison: Absorption, Fluorescence, ISC, Triplet State, and Quantum Yield of Singlet Oxygen

Metal-free porphyrins typically exhibit the presence of two internal protons and the lower symmetry of the macrocycle contribute to the splitting of the Q-bands; therefore, the spectrum of such compounds often contains four weaker Q-transitions. Upon coordination of Zn(II), the symmetry of the electronic system increases, and the spectrum of many Zn-porphyrins simplifies to two more intense Q-bands [6,46,71]. This has practical significance: more distinct Q-bands facilitate spectrophotometric monitoring of the photosensitizer’s concentration, photostability, and aggregation state.

From a therapeutic and diagnostic standpoint, Zn(II) porphyrins are often more convenient because the d10 configuration of Zn(II) does not create open-shell paramagnetic quenching channels characteristic of Cu(II), Fe(III), or Mn(III) porphyrins [126,130]. Unlike many redox-active metals, Zn(II) under physiological conditions generally does not participate in direct cyclic electron transfer but alters the ligand’s energy structure. Therefore, Zn-porphyrins can combine significant fluorescence with a high ability to form a triplet state, making them suitable for both photodynamic therapy and fluorescent monitoring of photosensitizer accumulation [16,121].

The ISC is a central process in PDT, since it is the triplet state of the photosensitizer that transfers energy to molecular oxygen to form ^1^O_2_ or participates in Type I electron or hydrogen atom transfer reactions [6,131]. In metal-free porphyrins, the efficiency of the ISC may be sufficient for PDT, but it depends more strongly on peripheral substituents, the solvent, aggregation, and the local microenvironment. In Zn(II) porphyrins, the central metal can optimize the ratio between the fluorescent channel and the triplet channel without driving the molecule into a state of complete non-radiative quenching [17,121,129].

Three parameters are important for the generation of singlet oxygen: the quantum yield of the triplet state, the triplet energy, and the lifetime of the triplet state. If the triplet energy is sufficient to sensitize O_2_, and the triplet lifetime allows for collisions with oxygen, the photosensitizer can operate effectively via a Type II mechanism [6,132]. Many Zn-porphyrins exhibit high ^1^O_2_ yields, especially when they are in the monomeric form and do not form dense H- or J-aggregates [56,121]. Nevertheless, one cannot draw a universal conclusion that a Zn-complex is always photodynamically more active than its metal-free analog. In some series of compounds, not only metalation but also charge, meso-substituents, conjugation, formulation type, and localization within the cell are decisive factors [13,16].

After discussing the photophysical parameters, it is necessary to move on to stability, since therapeutic efficacy depends on the preservation of the active monomeric form in the biological environment.

### 8.2. Chemical Stability, Photostability and Aggregation

Metal-free porphyrins can be quite stable; however, their internal N-H protons allow for protonation, deprotonation, tautomerism, and sensitivity to the acidity of the medium. In Zn-porphyrins, the central metal fixes the coordination geometry of the core, often resulting in a more well-defined molecular structure and reduced variability in spectral behavior [71,133]. However, Zn(II) coordination also introduces a new level of chemical regulation: axial coordination of water, histidine, pyridine, imidazole moieties, or protein donors can alter aggregation, solubility, localization, and photochemical activity [34,124].

Aggregation is one of the main causes of reduced photodynamic efficiency in porphyrins. The flat aromatic core is prone to π-π-stacking, especially in aqueous media, if the compound is hydrophobic or insufficiently charged. Both metal-free porphyrins and Zn-porphyrins can aggregate, but Zn(II), due to the greater planarity of the core and the possibility of axial coordination, can either promote or inhibit aggregation depending on the structure [34,56]. For example, axial ligands or bulky peripheral groups can reduce contact between macrocycles, maintain the monomeric state, and increase the ^1^O_2_ yield. Conversely, hydrophobic Zn-porphyrins without sufficient steric or charge protection can form inactive aggregates with accelerated non-radiative energy dissipation [56,133].

Photostability is also not a simple property determined exclusively by the presence of Zn(II). Photodegradation depends on oxygen, the solvent, light intensity, sensitizer concentration, local production of ROS, and the presence of biomolecular targets [134]. Some Zn-porphyrins exhibit good photostability and are suitable for prolonged irradiation, while others may undergo photobleaching, especially under conditions of severe oxidative stress. For PDT, this has dual implications: moderate photobleaching may limit post-treatment phototoxicity, but excessive photodegradation before the end of irradiation reduces the therapeutic effect [6,134].

Physicochemical differences become significant only when they alter cellular uptake, toxicity, and the selectivity of the photodynamic effect.

### 8.3. Biological Activity: Cellular Uptake, Localization, Dark Toxicity, Phototoxicity, and Selectivity

The biological activity of metal-free and Zn-porphyrins is determined not only by photophysics. Within the cell, a photosensitizer must pass through several selection stages: binding to plasma proteins or culture medium proteins, interaction with lipoproteins, penetration through membranes or endocytosis, distribution among organelles, and preservation of the photoactive form until irradiation [9]. Metal-free porphyrins may exhibit good phototoxicity but often suffer from hydrophobicity, aggregation, and slow clearance. Zn-porphyrins can demonstrate a better balance between fluorescence, cellular accumulation, and ROS generation; however, this is not characteristic of the entire class, but rather of specifically optimized structures [16,22,133].

Charge and amphiphilicity are often more important for cellular selectivity than the fact of metallation itself. Cationic porphyrins interact better with negatively charged bacterial membranes, mitochondria, or tumor cell membranes, whereas anionic and neutral derivatives may be more dependent on protein transport and lipoproteins [25,132]. Zn(II) may indirectly affect these processes through planarity, electron distribution, coordination with proteins, and changes in the hydrophobicity of the core. However, without properly selected peripheral substituents, Zn-porphyrin may remain poorly soluble and prone to nonspecific accumulation [13,56].

Dark toxicity is a critical parameter in the evaluation of clinical and preclinical photosensitizers. An important advantage of Zn(II) is that, unlike toxic heavy metals such as Cd and Hg, it exhibits low intrinsic toxicity and lacks the pronounced redox activity characteristic of Fe, Mn, and Cu in the corresponding porphyrin complexes [49,130]. Consequently, well-designed Zn-porphyrins often combine low dark toxicity with high phototoxicity upon irradiation [22,133]. Nevertheless, dark toxicity may increase as a result of cationic substituents, detergent-like membrane-disrupting properties, excessive lipophilicity, mitochondrial accumulation, or prolonged tissue retention [13,135].

Phototoxicity depends on the colocalization of ROS with susceptible cellular targets. When a metal-free or Zn-porphyrin localizes to mitochondria, PDT rapidly induces mitochondrial membrane depolarization, cytochrome c release, and apoptosis. When the compound accumulates in lysosomes, lysosomal membrane permeabilization and cathepsin-dependent cascades predominate. If the primary site of localization is the plasma membrane or the vascular endothelium, the contribution of necrosis, membrane lipid peroxidation, and vascular damage increases [13,77,133]. Therefore, Zn(II) does not automatically determine the mode of cell death but may influence the likelihood of specific subcellular localization through the coordination and physicochemical properties of the molecule [34,133].

Because Zn-porphyrins often retain appreciable fluorescence, they merit separate consideration as theranostic systems.

### 8.4. The Theranostic Potential of Zn-Porphyrins Compared to Their Metal-Free Analogs

Theranostics in PDT involves combining diagnostic imaging, monitoring of photosensitizer accumulation, and therapeutic irradiation. In this regard, Zn-porphyrins are particularly attractive because they can maintain sufficient fluorescence while effectively forming a triplet state to generate ROS [16,133]. Metal-free porphyrins can also fluoresce, although their spectra are often more complex, and their photophysical behavior is more strongly affected by protonation, aggregation, and the microenvironment [6,71].

The fluorescent signal from Zn-porphyrins can be used to assess cellular uptake, subcellular localization, biodistribution, and photobleaching during treatment [16,22]. This is particularly important for image-guided PDT, where not only high phototoxicity is required, but also the ability to determine whether a sufficient amount of photosensitizer has accumulated in the tumor tissue prior to irradiation. In this context, Zn(II) porphyrins occupy an intermediate position between highly fluorescent diagnostic dyes and heavy-metal porphyrins, in which the intersystem transition can be very efficient, although fluorescence is often suppressed [17,49,130].

Nanoparticle formulations significantly expand the theragnostic potential of Zn-porphyrins. Liposomes, micelles, polymeric nanoparticles, protein carriers, and porphyrin metal–organic frameworks can reduce aggregation, improve solubility, modify pharmacokinetics, and enhance passive or active tumor accumulation [39,51,56]. Nanoparticle formulations are also beneficial for metal-free porphyrins; however Zn-porphyrins additionally allow the metal center to be used as a coordination node, a sensing element, or a structural module in framework materials [34,39].

The comparison should conclude with a practical conclusion: the choice between the metal-free and Zn-coordinated forms depends on the desired balance between diagnostic performance, phototoxicity, and safety.

### 8.5. Summary: When Is Metal-Free Porphyrin Preferable, and When Does Zn-Porphyrin Have the Advantage?

Metal-free porphyrins are advantageous when a simple structure, lower coordination complexity, minimal risk of metal-dependent side interactions, and the possibility of subsequent metalation or modification are required. They also remain useful as basic models for comparing photophysics, aggregation, and biological activity [6,71]. However, for practical PDT, Zn-porphyrins are often preferred due to their better spectral resolution, favorable balance between fluorescence and triplet formation, high ^1^O_2_ generation potential, and suitability for theragnostic applications [16,22,133].

The optimal choice between a metal-free and a Zn-porphyrin compound cannot be made based solely on the name of the central element. It must be evaluated within the context of the entire structure-function system: central metal, peripheral substituents, charge, amphiphilicity, aggregation state, carrier, localization, light dose, tissue oxygen status, and the desired mechanism of cell death [13,51,135]. Zn(II) is particularly valuable when the research or clinical goal is to develop a photosensitizer with high Type II activity, fluorescence-controlled activity, low dark toxicity, and the potential for formulation optimization. Thus, Zn-porphyrins are not merely metallated analogs of metal-free porphyrins; they constitute a distinct design class of photosensitizers in which the central Zn(II) integrates photophysical, chemical, and biological properties into the PDT mechanism [16,22,34,133].

After comparing them with their metal-free counterparts, Zn-porphyrins should be more broadly classified among other metal porphyrin platforms.

## 9. Comparison with Other Metalloporphyrin’s

A comparative analysis should begin with Mg-based systems, as they provide a natural example of metal-dependent modification of the tetrapyrrole chromophore.

### 9.1. Magnesium Porphyrins and Chlorophyll-like Systems

Magnesium-containing tetrapyrrole pigments are a natural example of how the central metal can alter the function of the macrocycle without converting it into a strong redox catalyst. In chlorophylls, Mg(II) is coordinated within a chlorin rather than a fully porphyrin ring; consequently, such a system differs from classical synthetic porphyrins in the degree of macrocycle reduction, spectral profile, and biological role [32,136]. For photodynamic therapy, this example is important not because chlorophyll is itself a universal medicinal photosensitizer, but because it illustrates the principle that the central metal, hydrophobic environment, and peripheral substituents together determine light absorption, excited-state stability, and interaction with oxygen [115,136,137,138,139].

Mg(II)-porphyrins and chlorophyll-derived systems often exhibit intense absorption in the visible region and can efficiently populate excited states. However, their translational application is limited by chemical lability, photodegradation, demetalation, a tendency toward aggregation, and complex behavior in biological media [139,140]. Compared with Zn(II), magnesium generally stabilizes synthetic porphyrin complexes less effectively under physiological fluid conditions, and Mg-based systems are therefore more often regarded as model compounds or as scaffolds for chlorophyll-derived photosensitizers rather than as direct analogs of Zn-porphyrins [115,138].

From a mechanistic standpoint, Mg(II) does not confer pronounced biological redox activity on the macrocycle, but it does alter electron density, symmetry, and the energy distribution of excited states. This can influence the balance among fluorescence, internal conversion, ISC, and singlet oxygen generation [44,48]. In this respect, Mg-based systems are closer to Zn(II) porphyrins than to Fe-, Mn-, or Cu-porphyrins, yet they typically lag behind Zn-complexes in chemical stability, ease of modification, and predictability of pharmacological behavior [12,137].

From Mg-systems it is logical to move on to Pd- and Pt-porphyrins, which represent the opposite extreme—enhancement of triplet photophysics through the heavy-atom effect.

### 9.2. Palladium and Platinum Porphyrins

Pd(II)- and Pt(II)-porphyrins are classic examples of metalloporphyrins in which the central ion markedly enhances ISC through the heavy-atom effect. As a result, the triplet-state yield increases while fluorescence is often reduced or nearly quenched [44,82]. Such photophysics is beneficial for PDT, since the long-lived triplet state can efficiently transfer energy to molecular oxygen to generate singlet oxygen, or participate in electron transfer via a Type I mechanism [36,82].

Compared with Zn(II) porphyrins, Pd- and Pt-complexes may offer higher triplet efficiency and longer triplet lifetimes, but these advantages do not always translate directly into superior therapeutic outcomes. Excessively strong spin–orbit mixing can increase non-radiative losses, reduce diagnostic fluorescence, and alter the photobiological profile of the molecule [44,139]. Furthermore, heavy metals raise more demanding requirements for toxicological assessment, complex stability, clearance monitoring, and the absence of long-term tissue accumulation [83,140].

The particular significance of Pd- and Pt-porphyrins is also linked to oxygen sensing. Their phosphorescence is sensitive to quenching by oxygen, so these complexes can be used not only as photosensitizers but also as probes for evaluating tissue oxygenation [141,142]. In PDT, this opens the prospect of a theranostic approach: a single system can simultaneously report oxygen levels and trigger photochemical damage. For clinical PDT, however, Zn-porphyrins often offer a better balance among phototoxicity, fluorescence imaging, biocompatibility, and ease of further chemical design [12,143].

Beyond heavy metals, complexes with pronounced axial coordination deserve attention—in particular, Sn- and Al-porphyrins.

### 9.3. Tin and Aluminum Porphyrins

Sn(IV)- and Al(III)-porphyrins differ from Zn(II) porphyrins primarily in their coordination chemistry. Tin(IV) and aluminum(III) frequently form complexes with axial ligands, which alters geometry, polarity, solubility, aggregation tendency, and interactions with membranes or proteins [138]. Thanks to axial coordination, such photosensitizers can be functionalized with additional groups that enhance amphiphilicity, targeted delivery, or compatibility with nanoformulations [28].

Aluminum porphyrins and phthalocyanines demonstrate that a metal with relatively low redox activity can be highly effective. In fact, Al^3+^ and Mg^2+^ do not exhibit any intrinsic redox activity in biological systems. For Al-complexes, axial hydroxy, sulfo, or other polar moieties are particularly important, as they reduce aggregation and improve aqueous solubility [85]. By contrast, Zn(II) porphyrins tend to remain more planar and hydrophobic, making their efficacy more strongly dependent on peripheral substituents, carriers, and protein binding [12,28].

Sn(IV)-porphyrins offer an additional advantage: the high valence of the metal can stabilize axial ligands and generate sterically shielded structures that are less prone to π–π aggregation [30,144]. This is significant because aggregation reduces fluorescence, triplet yield, and ROS generation. At the same time, Sn-complexes require careful evaluation of toxicity, metal-binding stability, and metabolic fate. In summary, Sn- and Al-porphyrin systems demonstrate that the central metal can function not only as a photophysical modulator but also as an architectural element in molecular design [85,145].

Redox-active metals warrant separate consideration, as they can shift the porphyrin system from photosensitization toward more complex catalyzed redox chemistry.

### 9.4. Iron, Manganese, and Copper Porphyrins

Fe-, Mn-, and Cu-porphyrins differ fundamentally from Zn-, Mg-, Pd-, and Pt-porphyrins in that their central metals are capable of pronounced redox chemistry. Under biological conditions, such complexes can participate in electron transfer, peroxide conversion, superoxide dismutation, catalase- or peroxidase-like reactions, and catalysis of secondary ROS transformations [76,146]. Consequently, the same class of compounds can exhibit both photosensitizing and antioxidant or ROS-modulating properties, depending on structure, dose, medium, and light regimen [146,147].

Fe(III)-porphyrins are conceptually related to heme systems. Their biological relevance is high, but for PDT this creates a dual effect. On one hand, iron can promote radical processes, peroxide reactions, and the Type I component of photoaction. On the other hand, Fe-complexes often possess efficient non-radiative relaxation channels, shorter excited-state lifetimes, and lower singlet oxygen sensitization capacity compared with Zn- or Pd-porphyrins [76,129]. Moreover, iron’s involvement in reactions linked to ferroptosis or lipid peroxidation can complicate the interpretation of the photodynamic mechanism [144,146].

Mn(III)-porphyrins are frequently studied as catalysts of ROS transformations and as superoxide dismutase mimetics. In the PDT context, they can either amplify oxidative stress or, conversely, partially neutralize certain ROS, depending on the redox potential, charge, and localization of the complex [76,147]. This distinguishes them from Zn(II) porphyrins, where the central metal is not normally a direct redox participant in cellular damage. Copper porphyrins also represent a complex case: Cu(II) can quench excited states and reduce classical Type II efficiency, yet under certain conditions it may support electron-transfer and catalytic processes [44,76,148]. Thus, Fe-, Mn-, and Cu-porphyrins should be regarded not merely as alternative photosensitizers but as metal-redox-active platforms with potentially mixed pro-oxidant and antioxidant properties.

Following this analysis of different metals, general criteria by which a central ion is assessed for suitability in PDT can now be formulated.

### 9.5. Criteria for the Optimal Central Metal in PDT

The optimal central metal in a porphyrin photosensitizer must provide not a single isolated property but a coherent set of photophysical, chemical, and biological characteristics. Of primary importance are intense absorption within the therapeutic window, sufficient triplet-state energy for oxygen sensitization, a high quantum yield of ROS, controllable fluorescence for imaging, and adequate photostability [44,115,143]. If the metal quenches the excited state too efficiently, the photosensitizer loses PDT activity. If the metal excessively amplifies redox reactivity, the risks of dark toxicity, unpredictable biochemistry, and off-target oxidative stress increase [76,147].

From a biological perspective, the metal must support low dark toxicity, complex stability in plasma and within the cell, controlled protein interactions, low aggregation propensity, and compatibility with formulation strategies [12,28]. It must also avoid undesirable long-term accumulation or release of a toxic ion. In this context, Zn(II) occupies a special position: it is biologically abundant, generally redox-inert under physiological conditions, capable of enhancing fluorescence, and able to support efficient triplet-state formation without excessive metal-dependent catalytic toxicity [12,50,143].

Nevertheless, Zn(II) is not universally the best metal for every PDT scenario. For oxygen sensing or maximum triplet generation, Pd- and Pt-porphyrins may be more effective [82,146]. For axially functionalized amphiphilic systems, Sn(IV) or Al(III) may be advantageous [85]. For hypoxic tumors and Type I-oriented photochemistry, redox-active metals or hybrid nanoplatforms combining photosensitization with catalytic ROS conversion are sometimes worth considering [76,145,148]. The criterion of optimality therefore depends on the intended mechanism of action: classical singlet oxygen PDT, Type I PDT, theranostics, oxygen monitoring, immunogenic cell death, or combination therapy.

The comparison of metals thus leads to the conclusion that the optimality of the central ion depends on the specific therapeutic objective rather than on any single universal parameter.

### 9.6. Summary

Comparison of different metalloporphyrins shows that the central metal determines not only the spectral properties of the molecule but also the type of biochemical response following irradiation. Mg(II)-systems demonstrate the natural logic of tetrapyrrole photochemistry but have stability limitations. Pd(II)- and Pt(II)-porphyrins enhance triplet photophysics through the heavy-atom effect but may sacrifice fluorescence-based diagnostic convenience and require careful toxicological evaluation. Sn(IV)- and Al(III)-complexes are useful for axial design and aggregation control. Fe-, Mn-, and Cu-porphyrins unlock redox-dependent mechanisms but complicate the distinction among photosensitization, ROS catalysis, and antioxidant action [44,76,147].

Against this background, Zn(II) porphyrins occupy an intermediate yet practically very important position. They combine sufficiently favorable photophysics, high potential for singlet oxygen generation, useful fluorescence, and relative biological compatibility. Their mechanism of action is generally easier to interpret than that of redox-active metalloporphyrins, since Zn(II) predominantly does not participate in direct cellular redox cycling [12,143,147]. Zn-porphyrins therefore represent one of the most convenient platforms for the rational design of PDT agents, in which the central metal, peripheral substituents, formulation, and subcellular localization can be coordinated with the desired mechanism of photodynamic action.

The molecular properties of metalloporphyrin are realized in the tissue not in isolation but in the conditions of the tumor microenvironment, which can enhance or limit PDT.

## 10. Oxygen Dependence: Central Metal, Oxygen Consumption, and Type of ROS

Photodynamic therapy is fundamentally an oxygen-dependent modality, since most porphyrin photosensitizers, upon transition to the triplet state, transfer energy to molecular oxygen to generate singlet oxygen. The efficacy of PDT is therefore determined not only by photosensitizer concentration and light dose, but also by the local availability of O_2_ within tumor tissue [50,82,102,149]. The central metal in the porphyrin core influences this process indirectly: it modifies the triplet-state yield, lifetime, energy, susceptibility to non-radiative quenching, and the balance between Type II and Type I mechanisms [12,44,115]. Zn(II) porphyrins are characterized by a relatively favorable combination of fluorescence, triplet generation, and singlet oxygen photochemistry, whereas Pd(II)- and Pt(II)-complexes are more strongly shifted toward triplet photophysics through the heavy-atom effect, and Fe-, Mn-, and Cu-porphyrins may involve additional redox transformations [12,76,140].

Within the tumor, photodynamic oxygen consumption can occur faster than delivery through the vasculature. This is particularly relevant for dense or poorly vascularized tumors where diffusion limitations already exist [82,150]. If a Zn-porphyrin operates predominantly via a Type II mechanism, its efficacy may decline rapidly upon local O_2_ depletion. At the same time, this mechanism is well-controlled: the short radius of action of singlet oxygen confines damage to the region where photosensitizer, light, and oxygen are simultaneously present [102,115,149]. When redox-active metalloporphyrins or specially modified systems are used, the Type I component may be more pronounced; in this case, the generation of superoxide, hydrogen peroxide, hydroxyl radicals, or lipid radicals can potentially sustain phototoxicity at lower oxygen levels, albeit at the cost of greater complexity in biochemical interpretation [44,74,135].

The central metal influences oxygen dependence through a combined structure–photophysics–localization relationship rather than as an isolated parameter. Consequently, Zn-porphyrin PDT primarily follows the classical singlet oxygen model. To overcome the limitations of hypoxic tumors, strategies must include modifying the chemical formula, using fractionated light doses, incorporating oxygen carriers, or utilizing Type I photochemical mechanisms [138,150,151,152].

### 10.1. Hypoxia and Vascular Effects of PDT

Tumor hypoxia arises from uneven vascularization, chaotic vascular architecture, high interstitial pressure, rapid tumor growth, and high cellular oxygen consumption. For PDT, this has a dual significance. On one hand, low O_2_ levels directly limit ROS generation. On the other hand, PDT itself can damage the endothelium, induce vasoconstriction, microvascular thrombosis, edema, and secondary deepening of hypoxia [50,82,153]. The vascular component can be therapeutically useful, since disruption of the blood supply enhances tumor cell death, but it can also create survival zones for cells adapted to low O_2_ [150,154,155].

Metal dependence in this context manifests through photosensitizer localization and the ROS profile. If a metalloporphyrin accumulates preferentially in tumor endothelium or binds to plasma proteins, PDT may exhibit a strong vascular component. If a Zn-porphyrin or its nanoformulation penetrates tumor cells more effectively and localizes in mitochondria, lysosomes, or the ER, direct cellular damage may predominate over vascular effects [28,115,151]. The central metal does not independently determine vascular selectivity, but it influences planarity, charge, lipophilicity, axial coordination, aggregation, and protein binding—parameters that determine whether the molecule will remain in the vascular compartment or penetrate deeper into the tissue [12,24,28].

Hypoxia also activates HIF-1-dependent programs of survival, angiogenesis, metabolic reprogramming, invasion, and immunosuppression. PDT in a hypoxic tumor should therefore not be viewed merely as a brief photochemical event; it triggers a subsequent adaptive response from the microenvironment [135,150,156]. For Zn-porphyrins, this is particularly important, since their high singlet oxygen efficiency may be potent in well-oxygenated regions but diminished in deeply hypoxic zones. Rational approaches include fractionated illumination, lower light intensity, oxygen delivery, the use of MnO_2_-based, perfluorocarbon, or hemoglobin-like carriers, and combination of PDT with HIF-1 inhibitors or hypoxia-activated prodrugs [135,150,151,152,153].

### 10.2. Tumor Acidity: pH-Dependent Aggregation, Protonation, and Binding

Most solid tumors maintain a weakly acidic extracellular environment, resulting from glycolytic metabolism, lactate export, poor perfusion, and uneven CO_2_ removal. The pH of the tumor interstitium is typically lower than in normal tissues, while intracellular organelles maintain their own pH gradients: endosomes and lysosomes are considerably more acidic than the cytosol [157,158]. For porphyrin photosensitizers, this is significant because protonation of peripheral amino groups, charge changes, disruption of electrostatic interactions, and pH-dependent aggregation can radically alter uptake, fluorescence, ROS generation, and localization [28,157,158].

Zn(II) porphyrins may be particularly sensitive to the environment when they bear cationic, anionic, or pH-labile peripheral groups. At neutral pH, such molecules may interact preferentially with membranes or proteins, whereas in an acidic microenvironment enhanced protonation can increase water solubility, alter albumin binding, facilitate endosomal uptake, or, conversely, promote aggregation-induced quenching [24,159]. Aggregation is a critical issue: π–π stacking of porphyrin rings reduces effective triplet yield, quenches fluorescence, and decreases singlet oxygen production [65,96]. The pH-responsive design of Zn-porphyrins must therefore balance activation in the tumor environment with preservation of the monomeric, photochemically active state [157,160].

Contemporary pH-sensitive formulations exploit tumor acidity as a trigger for nanoparticle disassembly, charge reversal, unmasking of shielding groups, or photosensitizer release [157,160]. For Zn-porphyrins, this is promising: the central metal provides favorable photophysics, while peripheral chemistry or the carrier can ensure selective activation. Such systems have the potential to reduce dark toxicity and phototoxicity in normal tissues while increasing the concentration of the active monomeric photosensitizer in the more acidic tumor compartments [157,160].

### 10.3. Extracellular Matrix, Serum Proteins, and Lipoprotein Transport

The tumor microenvironment contains not only cells and vessels but also extracellular matrix, fibroblasts, macrophages, collagen fibers, glycosaminoglycans, albumin, lipoproteins, and other carriers. These components form a barrier to photosensitizer diffusion and simultaneously serve as pathways for their transport [24,28,161]. For porphyrins, binding to albumin, LDL, and HDL is especially important, since hydrophobic and amphiphilic tetrapyrroles often circulate not as free molecules but as complexes with proteins or lipoproteins [32,159,161]. Tumor cells frequently exhibit increased metabolic demand for lipids, and LDL-mediated delivery may therefore promote the accumulation of certain porphyrin photosensitizers [159,161].

The central metal influences protein and lipoprotein binding through macrocycle geometry, polarity, the presence of axial coordination, degree of aggregation, and electron distribution. Zn(II) porphyrins, being relatively planar and often hydrophobic, can associate extensively with protein hydrophobic pockets or lipid domains [28,32,115]. This has ambivalent consequences. On one hand, protein binding improves solubility, reduces nonspecific aggregation, and prolongs circulation. On the other hand, excessively tight binding may limit photosensitizer release within the tumor or alter its subcellular delivery [28,32,159].

The extracellular matrix can also influence the distribution of light and oxygen. Dense collagen regions increase diffusion resistance, raise interstitial pressure, and reduce nanoparticle penetration [24,162]. For Zn-porphyrin PDT, this means that high molecular phototoxicity does not guarantee in vivo efficacy if the formulation cannot overcome the stromal barrier. Promising strategies include albumin carriers, liposomes, polymeric nanoparticles, porphyrin MOF/COF systems, peptide targeting, and approaches that combine PDT with matrix remodeling or vascular normalization [24,151,153,162].

The tumor microenvironment should therefore be regarded as a filter through which molecular photophysics is converted into real therapeutic effect.

### 10.4. Summary: The Tumor Microenvironment as a Filter of Metal-Dependent Photochemistry

The tumor microenvironment transforms metalloporphyrin PDT from a simple photochemical reaction into a multilevel biophysical process. The central metal sets the potential of the molecule: absorption, fluorescence, triplet state, Type I/Type II balance, redox activity, and aggregation behavior. However, the microenvironment determines whether this potential will be realized within the tissue. Oxygenation regulates substrate availability for ROS; vasculature controls delivery and can itself be a PDT target; acidity modifies charge, aggregation, and activation; matrix and proteins govern transport, penetration, and bioavailability [24,28,50,82,102,149].

For Zn(II) porphyrins, this has a practical implication: they cannot be evaluated solely by their singlet oxygen quantum yield in solution. High photophysical efficiency must be paired with a formulation that maintains monomeric state, ensures tumor penetration, accounts for hypoxia, acidity, and protein binding, and directs the photosensitizer to the appropriate cellular or vascular target [28,115,151,160]. In well-oxygenated and accessible tumors, Zn-porphyrins can function as classical Type II photosensitizers. In hypoxic, dense, or highly stromal tumors, they are best combined with oxygen-modulating, pH-responsive, matrix-penetrating, or Type I-enhancing approaches [135,150,151,152,153].

The central metal and the tumor microenvironment are thus interdependent determinants of PDT. Zn(II) provides a favorable, relatively safe, and theranostically useful platform, but the ultimate therapeutic outcome depends on how well the molecular and carrier design matches the actual physicochemical conditions of the tumor. The next generation of Zn-porphyrin photosensitizers should be engineered not merely for maximum ROS generation, but for controlled performance within a specific microenvironment—defined by its oxygenation, pH, vascular architecture, protein transport, and stromal barrier [151,153,159,160,161,162].

Since the microenvironment can limit the action of Zn-porphyrins, the next logical step is an analysis of formulations capable of controlling their solubility, aggregation, and delivery.

## 11. Nanocarriers and Formulation Strategies for Zn-Porphyrin Photodynamic Therapy

The effectiveness of Zn-porphyrins in photodynamic therapy is determined not only by their own photophysical properties but also by the microenvironment in which the photosensitizer molecule finds itself after being introduced into a biological system. Many Zn-porphyrins are hydrophobic or amphiphilic compounds with a pronounced tendency toward π–π stacking. In an aqueous medium, this can lead to aggregation, reduced fluorescence, shorter lifetimes of excited states, and a decrease in the quantum yield of singlet oxygen [28,36,44,78]. Therefore, formulation is not a secondary pharmaceutical step but rather an integral part of the mechanistic design of PDT. A nanocarrier can maintain the monomeric state of porphyrin, alter its protein binding, increase local concentration within the tumor, ensure organelle targeting, and combine photodynamic action with chemotherapy, immunotherapy, photothermal therapy, or ferroptosis-targeted approaches [24,39,163,164,165].

It is advisable to begin the discussion of nanocarriers with liposomes and micelles, as they directly influence the membrane-like microenvironment of hydrophobic porphyrins.

### 11.1. Liposomes and Micelles

Liposomes are one of the most logical platforms for Zn-porphyrins, since the lipid bilayer is capable of accommodating hydrophobic porphyrin rings in a membrane-like environment. This environment partially mimics cell membranes, reduces the photosensitizer’s exposure to water, and may minimize inefficient aggregation [36,166,167]. This is particularly important for porphyrin derivatives: the monomeric form typically absorbs light better, transitions to the triplet state more efficiently, and sensitizes molecular oxygen more effectively, whereas aggregates often convert excitation energy into heat or non-radiative relaxation [44,79,166].

Liposomal systems also make it possible to regulate pharmacokinetics. Particle size, surface charge, cholesterol content, phospholipid saturation, and the presence of polyethylene glycol influence circulation in the blood, uptake by the mononuclear phagocytic system, interaction with albumin, and penetration into tumor tissue [163,165,168]. For Zn-porphyrin PDT, this means that a single molecule can have a different biological profile depending on whether it is freely bound to serum proteins, incorporated into a liposome, or presented as a lipid-anchored conjugate. Liposomes can serve as passive carriers or as active systems with ligands for tumor cell receptors, such as a folate moiety, an RGD peptide, an antibody, or an aptamer [24,167,169].

Polymer micelles perform a similar function, but their structure is based on the self-assembly of amphiphilic block copolymers. The hydrophobic core is capable of solubilizing Zn-porphyrin, while the hydrophilic crown stabilizes the particle in an aqueous medium [28,170]. Micelles are suitable for photosensitizers that are poorly soluble in physiological buffers. They can be pH-sensitive, redox-sensitive, or enzyme-sensitive, which allows for the release of porphyrin in an acidic endosomal–lysosomal environment, in a glutathione-rich cytosolic fraction, or in tumor tissue with elevated protease activity [39,50,170]. However, micelles have a critical association concentration; therefore, upon significant dilution in the blood, disintegration of the carrier and premature release of the photosensitizer are possible [170].

After discussing lipid and micellar systems, it is worthwhile to focus on polymer nanoparticles, which offer greater flexibility in terms of size control, release, and surface functionalization.

### 11.2. Polymer Nanoparticles

Polymer nanoparticles offer a wider range of designs: the photosensitizer can be physically encapsulated in a matrix, covalently attached to the polymer, or incorporated into the nanoparticle structure itself as a functional chromophore [28,164,171]. For Zn-porphyrins, it is important that covalent attachment can limit molecule leaching, reduce dark toxicity, and maintain a controlled release profile. An example of this approach is polymer-bound zinc protoporphyrin, which has been investigated as a tumor-targeted nanosensor for PDT and fluorescent imaging [172]. This type of design demonstrates a general principle: Zn-porphyrin can function not only as a photosensitizer but also as an optical component of a theragnostic system.

Materials based on PLGA, polymethacrylates, polyethylene glycol, polycaprolactone, or HPMA copolymers allow for the modification of size, charge, hydrophobicity, degradation rate, and biodistribution profile [168,171,173]. For PDT, it is important not only to deliver the molecule to the tumor but also to prevent it from remaining in an inactive aggregated state for too long. Therefore, an optimal polymer platform must combine high loading capacity with the porphyrin core’s photochemical accessibility to oxygen and light [28,168]. Excessively dense packing of chromophores in the matrix can cause concentration quenching; conversely, excessively loose packing can lead to nonspecific binding to membranes and serum proteins [79,166,171].

Polymer nanoparticles are also suitable for combination therapy. A single system can incorporate Zn-porphyrin, a chemotherapeutic agent, an antioxidant response inhibitor, an oxygen donor, a hydrogen peroxide decomposition catalyst, or an immunomodulator [24,39,50,164]. In such a design, the central Zn(II) acts as a photophysically active but predominantly redox-inert center; therefore, additional functions—such as the catalytic enhancement of Type I processes or glutathione depletion—are typically introduced via the nanomaterial, peripheral substituents, or a combined payload [39,174]. This fundamentally distinguishes Zn-porphyrins from Fe-, Mn-, or Cu-porphyrins, where the metal itself can participate in redox chemistry.

Coordination materials, in which metal centers and organic ligands can be part of the photodynamic platform itself, provide another level of control.

### 11.3. Metal–Organic Frameworks and Coordination Polymers

Porphyrin metal–organic frameworks (MOFs) and nanosystems derived from them are a particularly important platform for metal-dependent PDT. In such materials, porphyrin ligands can serve as structural nodes of the framework, while metal clusters act as components that determine porosity, stability, charge, and biological behavior [175,176,177]. As opposed to simple encapsulation, MOFs allow for the spatial separation of porphyrin chromophores, reduce self-quenching, and facilitate oxygen diffusion through the porous structure [175,176]. This is directly related to the PDT mechanism, since the generation of singlet oxygen requires simultaneous access to light, a photosensitizer, and molecular O_2_.

Zn-porphyrin or Zn-containing framework materials can perform several functions. First, they stabilize the porphyrin in an ordered state while preserving its photoactivity. Second, their porosity allows for the loading of additional molecules—chemotherapeutic agents, ferroptosis inducers, immunomodulators, or oxygen carriers [39,174,176]. Third, the MOF surface can be modified with polymer coatings, cell membranes, peptides, or antibodies to enhance biocompatibility and selectivity [175,176].

The relationship between the structural stability of MOFs and their biodegradation deserves special attention. A framework that is too stable may remain in the tissues for too long, whereas one that is too labile may degrade rapidly before reaching the tumor [175]. For PDT, controlled degradation is desirable after accumulation in the tumor microenvironment or following endocytosis. In the acidic environment of endosomes and lysosomes, some coordination frameworks may partially decompose, releasing photoactive porphyrin fragments or auxiliary components [39,174]. This is precisely why MOF platforms must be evaluated not only based on their size and photosensitizer loading but also on their photostability, biodegradation, clearance, dark toxicity, and behavior in the presence of serum proteins [175,176].

Alongside synthetic nanomaterials, biomolecular carriers are important, as they can combine drug delivery with natural biocompatibility and selective recognition.

### 11.4. Protein and Peptide Carriers

Protein carriers are a biologically understandable method for delivering porphyrins since many hydrophobic photosensitizers naturally bind to albumin, lipoproteins, and other plasma proteins [168,177]. Albumin has a long circulation time, the ability to transport hydrophobic molecules, and a tendency to accumulate in tumors due to increased vascular permeability and the active protein metabolism of tumor cells [168,177]. For Zn-porphyrins, albumin nanoparticles or conjugates can enhance aqueous dispersibility, reduce nonspecific aggregation, and mitigate dark toxicity [169,177].

Peptide systems make it possible to impart biological specificity to Zn-porphyrin. For example, RGD-like motifs can direct carriers to integrins, mitochondria-targeted lipophilic cations or peptides to mitochondria, and ligands for growth factor receptors to tumor cells with the corresponding phenotype [24,168,169]. Antibody-photosensitizer conjugates are an even more specific option: they can deliver a porphyrin or porphyrin-like photosensitizer to cells expressing a specific antigen, which is important for minimizing damage to normal tissues [168,178].

The main limitation of protein and peptide systems is the balance between selectivity and pharmacokinetic complexity. Large protein conjugates may penetrate dense tumor matrices more slowly, while peptides may degrade rapidly or be excreted by the kidneys [167,177]. In addition, the covalent attachment of Zn-porphyrin to a protein can alter its spectral properties: the proximity of aromatic amino acids, disulfide bonds, or other chromophores can affect fluorescence, intramolecular energy transfer, and oxygen access [167,177]. Therefore, protein-mediated delivery should be evaluated not only in terms of binding specificity but also in terms of the actual generation of ROS following binding to the cell.

Once the carrier ensures stability and delivery, the next challenge is to direct the photosensitizer to a specific organelle.

### 11.5. Organelle-Targeted Systems

Since singlet oxygen has a limited diffusion radius and a short lifetime in the cellular environment, the subcellular localization of Zn-porphyrin often determines the type of biochemical damage [24,80,83]. Nanocarriers can direct the photosensitizer to mitochondria, lysosomes, the endoplasmic reticulum, the Golgi apparatus, or the plasma membrane. Mitochondrial targeting is typically achieved through the use of lipophilic cations, peptides, or amphiphilic surface groups that respond to the membrane potential. Such systems can induce membrane depolarization, cytochrome c release, caspase activation, and apoptosis [24,80,83].

Lysosome-targeted carriers often use endocytosis as their primary route of entry. After accumulating in endosomes and lysosomes, PDT can cause lysosomal membrane permeabilization, the release of cathepsins, and secondary damage to mitochondria [24,80,83]. For Zn-porphyrins, this pathway is useful when the molecule itself has poor membrane permeability or exhibits strong protein binding. ER-targeted systems show promising results for inducing a response to misfolded proteins, calcium imbalance, and immunogenic cell death, yet require precise dose control, as excessive ER damage can rapidly progress to necrotic or mixed forms of cell death [24,80,83].

Different subcellular target profiles may be optimal for different tumors. Tumors with high anti-apoptotic dependence on the BCL-2 family may be more sensitive to mitochondrial PDT; cells with active autophagy or lysosomal biogenesis may be more sensitive to lysosomal damage; immunologically “cold” tumors may require ER stress and an immunogenic response [24,39,80]. Therefore, the Zn-porphyrin formulation should be designed not merely to “deliver more,” but to “deliver to the correct compartment.”

Thus, the formulation should be viewed not as an auxiliary pharmaceutical coating, but as a mechanistic regulator of Zn-porphyrin PDT.

### 11.6. Summary: Formulation as a Mechanistic Regulator of Zn-Porphyrin PDT

Nanocarriers alter the mechanism of Zn-porphyrin PDT at several levels. At the molecular level, they control aggregation, monomeric state, spectral properties, and oxygen access. At the cellular level, they determine the uptake pathway, subcellular localization, and the type of primary damage. At the tissue level, they influence circulation, tumor penetration, interaction with the matrix, serum proteins, and clearance [24,28,39,80,163,164,175]. Zn(II) itself provides a favorable photophysical basis—intense fluorescence, efficient triplet generation, and a high capacity for Type II photochemistry—but the formulation determines whether this potential is realized in a living system [44,79,172].

The most promising platforms are those that simultaneously address several issues: they increase solubility, prevent aggregation, maintain photochemical activity, ensure tumor- or organelle-specific targeting, reduce dark toxicity, and allow for controlled combination with other therapeutic modules [24,136,164,167,168,169,170,171,172]. In this sense, Zn-porphyrin is not merely a molecule that needs to be “encapsulated,” but an active photochemical center around which the entire nanomedical architecture is built. Rational formulation transforms Zn-porphyrin PDT from empirical light-dependent cytotoxicity into a controlled system in which the metal, macrocycle, carrier, target cell, oxygen status, and light dose function as a single mechanistic complex.

After the theoretical and formulation analysis, it is necessary to consider methods that allow for the experimental verification of metal-dependent mechanisms.

### 11.7. Experimental Approaches to Studying Metal-Dependent Mechanisms of PDT

Research into the metal-dependent mechanisms of photodynamic therapy requires a combination of photophysics, analytical chemistry, cell biology and systems-based bioanalytical methods. This is particularly important for Zn(II) porphyrins, since the central zinc ion not only stabilizes the tetrapyrrole core but also alters the electronic structure, aggregation state, fluorescent response, efficiency of the ISC, and ability to sensitize molecular oxygen [8,9,45,115]. Therefore, the experimental evaluation of Zn-porphyrin PDT cannot be limited to measuring overall phototoxicity. It is necessary to separately characterize the spectral properties of the photosensitizer, its chemical stability, the generation of ROS, intracellular localization, the type of cellular damage, the temporal dynamics of stress responses, and its behavior in vivo model systems [2,12,179,180,181]. This chapter reviews the main methods that allow one to distinguish the actual metal-dependent contribution from the effects of peripheral substituents, formulation, light dose, and biological model.

Any mechanistic investigation must begin with photophysical characterization, as otherwise it is impossible to separate the properties of the molecule from the cellular context.

## 12. Photophysical Characteristics

The initial stage of the study involves the characterization of the photophysical properties of the photosensitizer before its introduction into a cellular system. For Zn(II) porphyrins, a change in the Q-band profile compared to metal-free analogs is typical, reflecting a modification of the macrocycle’s electronic structure [44,45,115]. Comparing spectra in different solvents, buffers, serum, liposomes, or polymeric nanoparticles helps determine whether the molecule remains in its monomeric state under conditions close to biological ones [24,181].

Fluorescence spectroscopy is used to determine the emission maximum, quantum yield of fluorescence, photostability, and the potential of the photosensitizer for therapeutic applications. Zn(II) porphyrins often exhibit fairly strong fluorescence, allowing the photodynamic effect to be combined with visualization of cellular accumulation [12,115,182]. However, high fluorescence does not always directly correlate with high phototoxicity, since part of the absorbed energy may be lost through radiative or non-radiative channels rather than being transferred to the triplet state. Therefore, fluorescence data must be supplemented with an assessment of triplet states and singlet oxygen production [8,9].

Phosphorescence, laser flash photolysis spectroscopy, and transient absorption allow us to determine the lifetime of the triplet state, triplet-triplet transitions, and the efficiency of oxygen quenching. These parameters are most closely related to Type II PDT, since the formation of ^1^O_2_ depends on the energy matching between the triplet state of the photosensitizer and the ground triplet state of oxygen [8,9,119]. Photobleaching analysis, conducted at a controlled light dose, allows for the assessment of a compound’s chemical stability, its susceptibility to photooxidative degradation, and potential loss of activity during therapeutic irradiation [72,179].

Once the spectral and triplet parameters have been established, it is necessary to directly assess which ROS are formed during irradiation.

### 12.1. Detection of ROS

The evaluation of ROS is a central but methodologically complex element of PDT analysis. For the Type II mechanism, the direct or indirect detection of singlet oxygen is of paramount importance. The most specific physical approach is the detection of weak ^1^O_2_ phosphorescence at approximately 1270 nm; however, this method requires sensitive equipment and careful control of the solvent, oxygen conditions, and background signals [8,119]. In most biological studies, chemical or fluorescent probes are more commonly used, including Singlet Oxygen Sensor Green, DPBF, ABDA, and other ^1^O_2_ traps [8,183]. These results should be interpreted with caution, as the probes themselves may undergo photobleaching, react with other ROS, or alter the local microenvironment of the photosensitizer [99,183].

DCFH-DA, CellROX, H2DCFDA, and similar indicators are often used for a general assessment of oxidative stress. They are useful for comparing irradiation conditions; however, they do not allow for the unambiguous identification of a specific type of ROS [100,101]. Superoxide can be assessed using DHE or MitoSOX; however, the oxidation of these probes is not always strictly specific and requires confirmation by chromatography or control inhibitors [99,184]. Lipid peroxidation should be analyzed using C11-BODIPY581/591, malondialdehyde measurement, 4-hydroxynonenal, or mass spectrometric profiling of oxylipins [184,185].

To identify metal-dependent differences, it is important to combine several independent assays. For example, Zn(II) porphyrin, which effectively generates singlet oxygen in solution, may produce a mixed Type I/Type II profile in cells due to local hypoxia, contact with membranes, or aggregation in lysosomes [2,8,179].

The use of ROS absorbents such as sodium azide, histidine, mannitol, superoxide dismutase, catalase, or lipid peroxidation inhibitors can aid in the functional verification of the mechanism, but these reagents have their own toxicity and vary in their ability to penetrate cellular compartments [99,185].

Determining the type of ROS must be supplemented by an analysis of its localization, since the same particle can cause different effects depending on where it is formed.

### 12.2. Analysis of Subcellular Localization

Subcellular localization is one of the main factors determining the biochemical outcome of Zn-porphyrin PDT. Due to the short range of singlet oxygen, the primary targets are usually located near the site of photosensitizer accumulation [8,186]. Confocal fluorescence microscopy allows for the assessment of Zn-porphyrin accumulation in mitochondria, lysosomes, the endoplasmic reticulum, the Golgi apparatus, the plasma membrane, or the nucleus. To achieve this, colocalization with organelle markers such as MitoTracker, LysoTracker, ER-Tracker, Golgi-Tracker, Hoechst, or membrane dyes is used [12,182,186].

Quantitative assessment of colocalization should be interpreted with caution. Simple visual overlap of signals is not sufficient evidence of organellar localization. It is advisable to calculate Pearson or Manders coefficients, monitor spectral bleed-through, assess photobleaching, and distinguish true colocalization from random overlap in thick cellular regions [186,187]. Fluorescence lifetime imaging microscopy can provide additional information about the photosensitizer’s microenvironment, its aggregation state, and protein-membrane interactions, since fluorescence lifetime often varies depending on the polarity, viscosity, and proximity of quenchers [182,187].

Organelle fractionation can complement microscopy, especially when it is necessary to compare the accumulation of metal-free porphyrin, Zn-porphyrin, and other metal porphyrins in the same cell line. After isolating mitochondrial, lysosomal, microsomal, or nuclear fractions, the amount of photosensitizer can be determined by fluorimetry, spectrophotometry, or LC-MS [179,186]. This approach is useful for confirming imaging results; however, it requires verifying the purity of the fractions using protein markers and accounting for potential redistribution losses of the photosensitizer during cell lysis [186].

After localization, it is necessary to assess the functional outcome of PDT—organelle damage, viability, and the type of cell death.

### 12.3. Analysis of Cell Death and Functional Damage

Phototoxicity should not be evaluated using solely a single viability assay. MTT, resazurin, CCK-8, ATP-luminescence assays, neutral red, and crystal violet measure different aspects of cellular status: metabolic activity, redox potential, membrane integrity, adhesion, or biomass [179,188]. Following PDT, these parameters may diverge, as early mitochondrial dysfunction reduces the MTT signal prior to irreversible cell death, whereas adherent cells may remain on the surface but lose their clonogenic potential [2,186]. Therefore, for cancer models, clonogenic analysis, which assesses the long-term ability of cells to form colonies after photodynamic damage—is particularly important [188].

To classify cell death, the following methods are used: Annexin V/PI or Annexin V/7-AAD, caspase activity analysis, PARP cleavage, TUNEL, LDH release, measurement of mitochondrial membrane potential using JC-1, TMRE, or TMRM, as well as live-cell imaging [2,12,93]. In the case of Zn-porphyrins, localization in mitochondria is often associated with membrane depolarization, cytochrome c release, and caspase-dependent apoptosis, whereas lysosomal or plasma membrane damage may contribute to necrotic or mixed forms of cell death [2,12,93].

Autophagy, ER stress, ferroptosis-like processes, and immunogenic cell death should be evaluated separately. LC3-II, p62/SQSTM1, Beclin-1, and the use of autophagy flux inhibitors allow for distinguishing between the induction of autophagy and the inhibition of lysosomal degradation [115,189]. Markers such as CHOP, ATF4, XBP1 splicing, BiP/GRP78, and eIF2alpha phosphorylation indicate UPR activation [115,190]. For a ferroptosis-like response, C11-BODIPY, GPX4, GSH/GSSG, ACSL4, and sensitivity to ferrostatin-1 or liproxstatin-1 are informative [184,191]. For immunogenic cell death, assessment of calreticulin exposure, ATP release, HMGB1, and dendritic cell activation are used [123].

Classical cellular assays provide a localized picture, whereas omics approaches allow for the observation of the cell’s systemic response to photodynamic damage.

### 12.4. Omics Approaches

Transcriptomics, proteomics, redox proteomics, lipidomics, and metabolomics enable a shift from describing individual markers to a systematic analysis of the metal-dependent response to PDT. RNA-seq can detect the activation of genes involved in antioxidant defense, the UPR, inflammation, apoptosis, autophagy, metabolic reprogramming, and interferon- or NF-κB-dependent pathways [112,180]. Comparing Zn-porphyrin with its metal-free analog at the same light dose can reveal whether the central metal alters not only the severity of damage but also the nature of the cellular response [180,192].

Proteomics and phosphoproteomics are particularly useful for analyzing early events following PDT, when the transcriptional response has not yet fully developed. They allow for the detection of changes in proteins involved in mitochondrial respiration, the cytoskeleton, the proteasome, heat shock, the repair system, and the MAPK, PI3K-AKT, or JNK signaling cascades [180,189,191]. Redox proteomics, including the analysis of oxidized cysteines, protein carbonylation, or methionine modifications, enables the direct mapping of protein targets of photooxidation [185,192].

Lipidomics is particularly important when a photosensitizer is localized in membranes or when a ferroptosis-like response is being studied. It allows for the detection of oxidized phospholipids, changes in cardiolipin, sphingolipids, cholesterol, and aldehyde products of peroxidation [184,191]. Metabolomics can reveal depletion of NADPH, GSH, and ATP, as well as disruptions in the tricarboxylic acid cycle, glycolysis, and amino acid metabolism following PDT [180,192]. For reliable interpretation of omics data, time series, dark controls, light controls without a photosensitizer, control of photosensitizer concentration, and independent functional validation of key pathways are required [180,192].

The final stage of experimental validation involves in vivo models, where photophysics, pharmacokinetics, the microenvironment, and dosimetry interact simultaneously.

### 12.5. In Vivo Models and Translational Studies

In vivo evaluation of Zn-porphyrin PDT should encompass not only tumor volume reduction but also pharmacokinetics, biodistribution, tumor accumulation, clearance, phototoxicity in normal tissues, and histological evidence of the mechanism of action [102,179]. Xenograft models allow for rapid comparison of photosensitizers, light doses, and formulations, but they do not fully replicate the immune response and microenvironment of a human tumor. Orthotopic and immunocompetent models are better suited for analyzing vascular effects, hypoxia, inflammation, and immunogenic cell death [102,123].

The biodistribution of Zn-porphyrins can be assessed by fluorescence imaging, ex vivo organ spectrofluorimetry, LC-MS, or histological analysis of tissue sections. For nanoparticle-based formulations, it is important to distinguish the signal from the photosensitizer itself from that of the carrier, since their distribution may not coincide after the molecule is released [24,102,182]. Histology, TUNEL, and immunohistochemistry for cleaved caspase-3, γH2AX, 4-HNE, HMGB1, CD31, HIF-1alpha, or immune cell markers help link the macroscopic therapeutic effect to cellular mechanisms [94,102,122].

Standardization of the light dose is a critical requirement for translational research. It is necessary to specify the wavelength, power, fluence rate, total fluence, irradiation geometry, light penetration depth, and the interval between photosensitizer administration and irradiation [102,179]. Without these parameters, it is impossible to compare the efficacy of different Zn-porphyrins or draw conclusions about the role of the central metal. Consequently, an optimal experimental design should combine photophysical characterization, quantitative analysis of ROS, organelle localization, functional cellular assays, systems omics analysis, and validated in vivo models. It is precisely this multilevel approach that allows us to view Zn(II) not as a formal structural element, but as a regulator of the photodynamic action mechanism.

The experimental data must be evaluated from a translational perspective—that is, in terms of the realistic possibility of transforming Zn-porphyrin systems into controllable clinical tools.

## 13. Clinical and Translational Significance

Porphyrin-mediated photodynamic therapy (PDT) is no longer merely an experimental photochemical concept. It is used or actively investigated in oncology, dermatology, ophthalmology, dentistry, the treatment of localized infections, and the development of antimicrobial materials [16,102,104,190]. Its translational appeal lies in the locality of action: the photosensitizer accumulates in tissue, after which light defines the spatial and temporal zone of activation. For Zn-porphyrins, this general advantage is complemented by tunable photophysics, intense fluorescence, high singlet oxygen generation capacity, and the relative redox inertness of Zn(II) under physiological conditions [6,12,32,115]. However, the clinical success of such systems depends not only on the quantum yield of ROS but also on pharmacokinetics, solubility, selectivity, phototoxicity, standardization of light dosimetry, and reproducibility of synthesis [24,39,103,190].

Before assessing the future potential of Zn-porphyrins, it is necessary to briefly define the current place of porphyrin-based PDT in medicine.

### 13.1. Current Status of Porphyrin-Based PDT in Medicine

In clinical medicine, the most established applications of PDT remain localized precancerous and tumor lesions, superficial or endoscopically accessible tumors, dermatological conditions, certain ophthalmological states, and antimicrobial photodynamic inactivation [16,102,104,193]. Classical porphyrin and chlorin photosensitizers have demonstrated that PDT can be effective when the margins of the lesion can be illuminated in a controlled manner and the photosensitizer has sufficient selectivity for the pathological tissue [102,149,190]. In dermatology and superficial oncology this is particularly relevant, as light is easily delivered to the target and local toxicity can be controlled.

In oncology, PDT occupies a specific niche: it does not always compete with surgery, chemotherapy, or immunotherapy as a systemic modality, but it can be valuable for local tumor control, palliative tumor debulking, endoscopic treatment, combination regimens, and stimulation of antitumor immune responses [98,123,190,193,194,195]. For porphyrin systems, it is important that the mechanism of damage is not reducible to a direct cytotoxic effect. PDT can simultaneously damage tumor cells, the vascular component, and stromal barriers, and can initiate immunogenic cell death [98,123,193,196].

Antimicrobial PDT is being considered as a strategy that is independent of classical mechanisms of antibiotic resistance. Cationic porphyrins and metalloporphyrins can bind to bacterial membranes, biofilms, or fungal cell walls, after which local ROS generation damages membranes, proteins, nucleic acids, and the biofilm matrix [24,104]. For Zn-porphyrins, this direction is important owing to the combination of fluorescence-based localization monitoring with photodynamic activity, although clinical implementation requires optimization of carriers, light penetration depth, and minimization of tissue staining [12,105].

Against the backdrop of existing clinical experience, the advantages of Zn-porphyrins as a platform for further translation can be considered separately.

### 13.2. Advantages of Zn-Porphyrins as a Translational Platform

Zn-porphyrins possess several properties that make them particularly promising for mechanism-driven PDT. First, complexation with Zn(II) often increases macrocycle symmetry, modifies the Q-bands, stabilizes the planar structure, and can improve fluorescence imaging [6,12,32,115]. This is important for theragnostics, where a single molecular platform serves both diagnostic and therapeutic purposes. Fluorescence allows the accumulation of the photosensitizer, its intracellular distribution, and lesion margins to be assessed, and potentially enables monitoring of photobleaching during illumination [12,115,197].

Second, Zn(II) is a d^10^ ion and generally does not participate in strong redox cycling within cells. Zn-porphyrins therefore tend to act as photosensitizers in which the principal damaging mechanism is linked to energy transfer from the triplet state to molecular oxygen and the generation of singlet oxygen [6,32,44]. This relative redox inertness distinguishes Zn-porphyrins from Fe-, Mn-, or Cu-porphyrins, for which the metal center can alter the ROS profile, catalyze peroxide transformations, or act as a ROS modulator [32,44,74].

Third, the Zn-porphyrin core combines conveniently with peripheral substituents, targeting ligands, polymeric carriers, liposomes, micelles, albumin systems, and porphyrin metal–organic frameworks [24,28,39,198,199]. This makes Zn-porphyrins not merely a distinct class of molecules but a modular platform in which the central metal establishes the photophysical foundation while peripheral chemistry and formulation determine biodistribution, cellular uptake, and the therapeutic window.

The translational attractiveness of Zn-porphyrins must, however, be assessed together with their limitations, which often determine real in vivo efficacy.

### 13.3. Limitations: Solubility, Aggregation, Selectivity, and Oxygen Dependence

The most important barrier to the translation of Zn-porphyrins is not toxicity alone but physicochemical controllability. Many porphyrins are hydrophobic, prone to aggregation in aqueous media, and prone to binding with serum proteins or membranes [24,28,103]. Aggregation reduces fluorescence, shortens effective excited-state lifetimes, and decreases singlet oxygen generation [6,24]. A molecule that demonstrates high photodynamic activity in an organic solvent may therefore prove significantly less active in serum, cell culture medium, or tumor tissue.

Another problem is insufficient selectivity. Passive accumulation via the enhanced permeability of tumor vessels is inconsistent and depends on tumor type, vascularization, stromal pressure, and nanoparticle size [28,39,98]. Active targeting with antibodies, peptides, or receptor ligands can improve specificity, but simultaneously complicates synthesis, quality control, immunogenicity, and scale-up [39,199]. For Zn-porphyrins, the optimal translational strategy should combine high photoactivity with pharmacological simplicity, not merely with the most complex possible nanoconstruct.

The oxygen dependence of PDT remains a fundamental limitation. Singlet oxygen requires the presence of molecular oxygen in the illuminated zone, whereas tumors frequently contain hypoxic regions [98,193,196]. Zn-porphyrins operating predominantly via a Type II mechanism can be highly effective in well-oxygenated tissue but lose activity in the hypoxic tumor core [6,32,199]. This explains the interest in combining Zn-porphyrins with oxygen-generating materials, hydrogen peroxide catalysts, perfluorocarbon carriers, or strategies that enhance the Type I contribution [39,199,200].

### 13.4. Safety Aspects: Dark Toxicity, Photosensitivity, and Clearance

The clinical safety of a photosensitizer is defined by the difference between dark toxicity and light-induced toxicity. For Zn-porphyrins, low cellular damage in the absence of illumination, stability in blood, absence of unwanted redox cycling, and sufficiently rapid clearance after therapy are all desirable [12,24,103]. Dark toxicity can arise through membrane destabilization, mitochondrial accumulation, DNA binding, or nonspecific protein interactions, even without photochemical activation [103,194].

Prolonged cutaneous photosensitivity is a classic limitation of many porphyrin photosensitizers [102,103,148]. If the molecule or its aggregates persist in the skin for extended periods, the patient requires light protection for days or weeks. From a Zn-porphyrin design perspective, this means that high lipophilicity and strong membrane binding are not always advantageous. A balance is required between accumulation in pathological tissue, a sufficient window for illumination, and subsequent clearance [24,28,103].

Metal-specific toxicity is generally lower for Zn(II) than for some redox-active metals, but this does not automatically confer safety on the entire molecule. Toxicity is determined not by the metal alone but by the complete structure of the photosensitizer, counter-ions, synthetic impurities, the nanocarrier material, and photodegradation products [24,39,197]. Translation therefore requires not only cellular MTT or resazurin assays but also hemocompatibility testing, immunological evaluation, pharmacokinetic profiling, tissue histology, and analysis of long-term accumulation [39,103,194,198].

Safety and efficacy can only be demonstrated under conditions of standardized synthesis, pharmacokinetic monitoring, correct dosimetry, and mechanistic validation.

### 13.5. Requirements for Translation: Synthesis, Pharmacokinetics, Light Dosimetry, and Mechanistic Validation

The transition from a laboratory Zn-porphyrin to a candidate for preclinical or clinical development requires reproducible synthesis, purity control, stable formulation, and scalability [24,39,197]. Minor variations in the degree of metallation, free-base impurities, residual solvents, monomer-to-aggregate ratio, or nanoparticle size can substantially alter photoactivity and toxicity [6,24,28]. The analytical package should therefore include NMR, HRMS, elemental analysis or ICP-MS for the metal, UV-Vis spectroscopy, fluorescence measurements, singlet oxygen determination, stability in buffers and serum, and—for nanoformulations—DLS, zeta potential, and morphological characterization [6,24,39].

Pharmacokinetics is a central translational parameter. It is necessary to know when the photosensitizer reaches maximum accumulation in the tumor or pathological tissue, when its concentration in normal tissues decreases, and at which point illumination yields the best therapeutic window [28,39,103]. For Zn-porphyrins, fluorescence can aid visual or quantitative monitoring of biodistribution, but the fluorescent signal does not always directly equal photodynamic activity, particularly when aggregation or protein binding is present [12,117,197].

Light dosimetry is as important as drug dose. The PDT effect depends on wavelength, power density, total light dose, illumination geometry, light penetration into tissue, oxygenation, and photobleaching [102,190,194]. Comparing Zn-porphyrins with other photosensitizers requires more than reporting concentration and incubation time alone. Optical parameters must be standardized, the actual dose in tissue or a model system must be measured, and phototoxicity must be evaluated in relation to the level of accumulation [197,201].

Mechanistic validation should confirm that the central metal genuinely determines the response rather than serving merely as a structural label. It is therefore useful to compare a Zn-porphyrin with its metal-free analog, analogs bearing other metals, demetalated or axially modified forms, and with identical carriers and light regimens [6,32,44,76]. Such a design allows the contribution of Zn(II) to photophysics to be separated from the contributions of charge, lipophilicity, aggregation, and subcellular localization.

The combined assessment of advantages and limitations makes it possible to define the place that Zn-porphyrins may occupy in the next generation of photodynamic therapy.

### 13.6. Summary: The Place of Zn-Porphyrins in the Next Generation of PDT

The translational prospect of Zn-porphyrins lies in the possibility of their serving not merely as phototoxic dyes but as rationally designed theranostic agents. Their central metal provides a favorable photophysical foundation, peripheral substituents regulate solubility and biodistribution, and carriers or targeting groups direct the molecule to the appropriate tissue or organelle [12,24,28,39]. The most promising systems are not universal Zn-porphyrins for all tumors but context-specific ones: for superficial lesions, endoscopically accessible tumors, fluorescence-guided local therapy, antimicrobial inactivation, and combination regimens with immunotherapy or nanomedicine approaches [16,39,193,199,202].

The clinical value of Zn-porphyrins is therefore defined not by any single characteristic but by the coherent alignment of structure, photochemistry, biological localization, safety, and technological reproducibility. Zn(II) at the center of the porphyrin macrocycle is a useful engineering element, but the ultimate therapeutic outcome depends on the entire molecular and pharmaceutical architecture. Future translation of Zn-porphyrin PDT must therefore rest on an integrated framework: metal center—peripheral chemistry—carrier—target organelle—ROS type—light dosimetry—clinical scenario [6,24,194,197,198,199,202].

## 14. Prospects for Development

The development prospects begin with rational design, as it is this that allows the central metal, substituents, localization, and ROS type to be linked into a unified system.

### 14.1. Rational Design of Metal-Centered Porphyrin Photosensitizers

Future progress in porphyrin PDT must move from empirical molecule selection toward the rational design of photosensitizers in which the central metal, peripheral substituents, charge, hydrophobicity, axial ligands, and formulation are treated as a unified functional system [16,32,102,201]. For Zn(II) porphyrins, this approach is particularly promising because Zn(II) combines redox inertness under physiological conditions with a pronounced influence on the electronic structure of the macrocycle, fluorescence, triplet state, and singlet oxygen generation [28,73,115]. The central zinc ion should therefore be regarded not merely as a stabilizing element of the porphyrin ring but as a regulator of the balance among the diagnostic, photochemical, and biological properties of the molecule.

A key prospect is the creation of Zn-porphyrin series with controlled red- and near-infrared-shifted absorption, reduced aggregation, and predictable organellar localization [24,32,203]. Peripheral cationic groups can enhance interactions with mitochondria and bacterial membranes, while amphiphilic or peptide fragments can facilitate membrane penetration, albumin binding, or receptor-mediated transport [39,174,200]. Importantly, optimizing a single parameter does not guarantee therapeutic success: a photosensitizer with a high singlet oxygen quantum yield may perform poorly in vivo owing to aggregation, poor tumor accumulation, or rapid clearance [175,200,204].

Rational design must also take into account the type of cellular response. When the goal is local tumor destruction, compounds with high phototoxicity and mitochondrial or lysosomal localization are appropriate [96,115]. When the therapeutic strategy aims at immune activation, systems that induce immunogenic cell death, release DAMP signals, and promote controlled inflammation may be preferable [65,205,206]. For hypoxic tumors, combining Zn-porphyrins with oxygen carriers, H_2_O_2_-decomposing catalysts, or components that shift the mechanism toward Type I reactions is a relevant approach [199,200,201,202,203,204,205,206,207].

### 14.2. Transition from Empirical PDT to Mechanism-Driven Therapy

The most important conceptual shift in the next generation of PDT is the transition from the generic statement “photosensitizer + light + oxygen” to mechanism-driven therapy, in which the molecular structure, photophysics, localization, ROS type, and cell death pathway are linked in a single cause-and-effect model [2,16,102]. For Zn-porphyrins, such a model must explain why the same basic porphyrin platform can function as a fluorescent probe, a mitochondrial photosensitizer, a nanoparticle component, or a theranostic module [24,73,175].

Mechanism-driven PDT requires standardization at several levels of analysis. At the first level, absorption spectra, fluorescence, photostability, aggregation, triplet properties, and singlet oxygen generation must be measured under conditions as close as possible to the biological environment [32,74,204]. At the second level, subcellular localization must be established, since the same ROS profile can produce different consequences in mitochondria, the ER, lysosomes, or the plasma membrane [96,115,202]. At the third level, not only short-term phototoxicity but also clonogenic survival, immunogenicity, vascular effects, and the potential for recurrence must be assessed [37,205,206].

A promising direction is the creation of “mechanism maps” for metalloporphyrin families. In such maps, the central metal, peripheral structure, charge, formulation, and light regimen are linked to the dominant ROS type, organellar target, and cellular response [16,177,208]. For Zn(II) porphyrins, this will allow the effects of the metal itself to be separated from those of substituents and the carrier. This approach is important for translation, as a clinically relevant agent must not simply be photochemically active but must be reproducible, safe, dosable, and mechanistically understood [37,102,175].

### 14.3. Zn-Porphyrins as Theranostic Platforms

One of the strongest prospects for Zn-porphyrins is their use as theranostic platforms combining fluorescence imaging, photodynamic action, biodistribution monitoring, and treatment response assessment [24,73,174]. Zn(II) coordination frequently sustains intense fluorescence of the porphyrin core, enabling the accumulation of the photosensitizer in cells or tissues to be tracked prior to irradiation [28,71]. This is particularly important for personalized PDT, where the therapeutic light dose can be adjusted according to actual drug accumulation, tumor depth, and local oxygen status [37,174].

In the future, Zn-porphyrin systems may be integrated with nanoplatforms for multimodal diagnostics. Porphyrin metal–organic frameworks, polymeric nanoparticles, liposomes, and protein carriers can combine the photosensitizer with contrast agents, chemotherapeutic drugs, immunomodulators, or hypoxia-activated components [24,160,178,208,209]. Such integration can simultaneously address multiple challenges: improving solubility, reducing aggregation, enhancing tumor accumulation, protecting the photosensitizer in the bloodstream, and ensuring controlled release in the pathological microenvironment [24,174,175].

At the same time, theranostics requires caution. Strong fluorescence does not always correlate with high photodynamic efficiency, since radiative relaxation can compete with ISC and triplet generation [32,72]. Future Zn-porphyrin platforms must therefore optimize the balance between the diagnostic signal and therapeutic photochemistry. The best candidates may be systems in which fluorescence is sufficient for imaging without reducing the efficiency of Type II or mixed Type I/Type II reactions [28,177,207].

### 14.4. Combination Strategies: Chemotherapy, Immunotherapy, Ferroptosis, and Hypoxia

Combination therapy is one of the principal routes to enhancing the efficacy of Zn-porphyrin PDT. PDT can locally damage tumor cells, vasculature, and stromal structures, but its outcome is limited by light penetration depth, oxygen dependence, and the tumor’s capacity to recover from sublethally stressful conditions [37,102,197]. Combination with chemotherapy can increase cytotoxicity when photodynamic damage disrupts membrane transport, mitochondrial function, or repair systems [160,178]. Nanoformulations are particularly well suited for such approaches, as they permit co-delivery of the photosensitizer and the drug component within a single particle [24,174,175].

Combination of PDT with immunotherapy is attracting considerable interest. Photodynamic damage can promote calreticulin exposure, release of ATP, HMGB1, heat-shock proteins, and oxidized lipids, thereby activating dendritic cells and antigen presentation [65,205,206,207,208,210,211]. However, PDT alone does not always overcome the immunosuppressive tumor microenvironment. Combining Zn-porphyrin photosensitizers with checkpoint inhibitors, adjuvants, STING agonists, or macrophage reprogramming strategies is therefore promising [65,206,209]. In this context, the central metal exerts an indirect influence through the ROS profile, localization of damage, and type of cell death.

Another promising direction is the combination of PDT with ferroptosis-inducing strategies. Although Zn(II) itself is not an iron-like redox-active ion, Zn-porphyrin PDT can initiate lipid peroxidation, depletion of antioxidant systems, and membrane damage, creating conditions for a ferroptosis-like response [127,197,204]. Adding iron-containing nanocomponents, GPX4 inhibitors, or agents that disrupt glutathione metabolism can amplify this pathway, but requires careful control of systemic toxicity [198,206].

For hypoxic tumors, future strategies should incorporate oxygen-generating, oxygen-storing, or Type I-oriented formulations [206,207,210]. These may include porous MOF platforms, perfluorocarbon carriers, catalase-like systems, MnO_2_ components, or redox-active auxiliary modules [153,206,207,210]. For Zn-porphyrins, such combinations are particularly important, since their classical advantage—efficient singlet oxygen generation—can become a limitation in environments with low partial pressure of O_2_ [204,206].

### 14.5. Computational Design and Artificial Intelligence

Computational methods are becoming an indispensable part of the future design of porphyrin photosensitizers. Quantum-chemical calculations, TD-DFT, molecular dynamics, modeling of membrane interactions, and machine learning can help predict absorption spectra, excited-state energies, aggregation propensity, protein and membrane interactions, and potential phototoxicity [16,209,212,213]. For Zn-porphyrins, this is particularly valuable, as minor changes in peripheral substituents or axial coordination can substantially alter solubility, planarity, electronic structure, and biological behavior.

A promising direction is the creation of integrated workflows in which computational screening selects molecules on the basis of multiple criteria: absorption within the therapeutic window, high probability of ISC, sufficient triplet energy for oxygen sensitization, low aggregation, acceptable synthetic accessibility, and predicted biocompatibility [16,209,213]. Machine learning can also help reveal non-obvious relationships between structure, formulation, and biological outcome, provided that training sets contain not only photochemical parameters but also data on localization, light dose, oxygen status, and type of cell death [213,214].

Computational design should not, however, replace experimental validation. The multilevel nature of the PDT mechanism is critical: the molecule, nanoformulation, cell, tissue, tumor microenvironment, and light delivery simultaneously shape the final effect [37,102].

Rational design therefore requires simultaneous control of the central metal, peripheral substitution, formulation and biological context rather than optimization of any single photophysical parameter in isolation (Figure 4). The most productive approach will therefore be a closed-loop cycle of prediction—synthesis—photochemical validation—cellular testing—in vivo verification—model update [209,212,213]. Precisely such a cycle can shorten the path from a large Zn-porphyrin library to a small number of genuinely translationally promising candidates.

### 14.6. Summary of Prospects

The future of Zn-porphyrin PDT lies not in simply generating yet more photosensitizers but in building mechanistically driven platforms. Central Zn(II) should be regarded as part of a multifactorial system in which the metal determines the electronic structure, peripheral substituents regulate bioavailability and localization, formulation controls delivery, and light dose directs the biological outcome. The most promising systems will be those in which photochemical activity is combined with controlled organellar action, imaging capability, adaptation to hypoxia, and the ability to trigger a beneficial immune response [24,177,204,206].

In the broader context, the central metal in the porphyrin scaffold is one of the keys to the transition from classical PDT to precision photomedicine. Zn-porphyrins can occupy an important position between simple organic photosensitizers and complex multicomponent nanoplatforms. Their advantage lies in the combination of relative biocompatibility, powerful photophysics, fluorescence potential, and chemical modifiability. Future development of Zn-porphyrin systems should therefore rest on the integration of biophysics, synthetic chemistry, cell biology, immunology, nanotechnology, and computational design.

**Figure 5:**
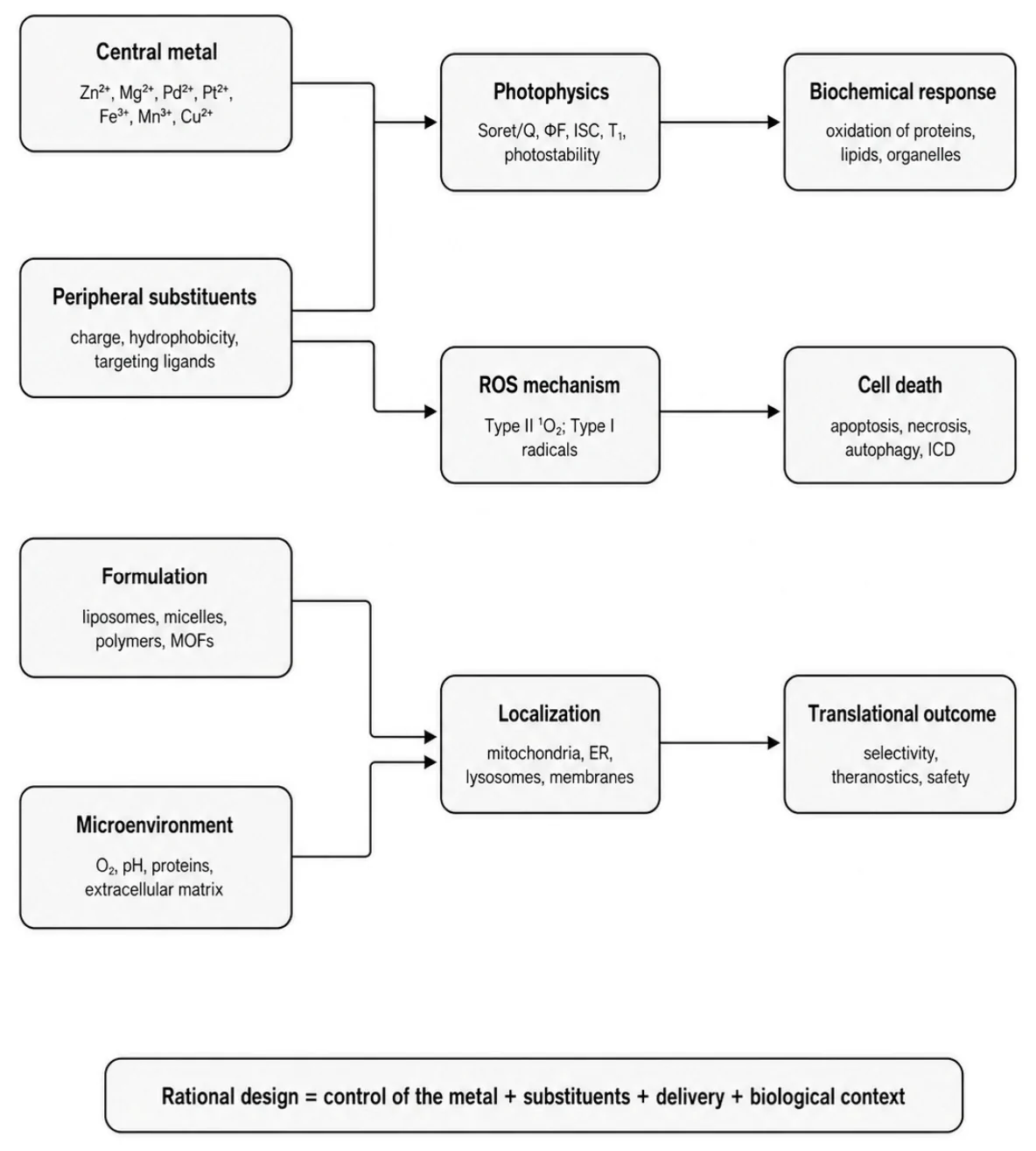
Structure mechanism relationship in metalloporphyrin PDT.

Following the sequential analysis of structure, photophysics, biochemistry, formulations, and translation, the principal conclusions must now be synthesized in Section 15 (Figure 5).

## 15. Discussion

It is appropriate to start the discussion with the main concept of the review: the central metal combines molecular structure with photochemical and biological results.

### 15.1. The Central Metal as an Integrator of Structure, Photophysics, and Biochemical Action

The material in the previous sections shows that the central metal in the porphyrin core cannot be regarded as a secondary structural element. In photodynamic therapy, it acts as an integrator between the molecular structure of the photosensitizer, its spectral properties, its ability to form a triplet state, the type of ROS, subcellular localization, and the final biological response [7,12,197,215]. For classical organic photosensitizers, the importance of peripheral substituents, hydrophobicity, charge and formulation is often emphasized. However, in metal porphyrins, it is the coordination center that alters the electron density of the macrocycle, the symmetry of the molecule, the possibility of axial coordination, aggregation, and interaction with proteins or membranes [24,214]. Therefore, the central metal does not act in isolation but as a factor that switches the same porphyrin platform between different mechanistic modes of PDT.

This perspective is important for interpreting experimental data. If two porphyrin photosensitizers have similar peripheral groups but different central metals, they cannot automatically be compared solely on the basis of dark toxicity or IC_50_ following irradiation. It is necessary to compare absorption spectra, fluorescence, singlet oxygen quantum yield, photostability, aggregation, cellular accumulation and organelle targeting [6,7,12]. Only such a multi-level approach allows us to understand whether a metal enhances photodynamic efficiency or, conversely, promotes non-photoproductive relaxation.

Once the integrative role of the metal has been defined, it is necessary to clarify the intermediate position of Zn(II) between metal-free and heavy-metal photosensitizers.

### 15.2. Zn(II) Porphyrins Between Metal-Free and Heavy Metal Photosensitizers

The unique feature of Zn(II) porphyrins lies in the combination of two properties: the relative biological compatibility of Zn(II) and the ability of zinc coordination to enhance the photophysical behavior of the porphyrin chromophore. Compared with metal-free porphyrins, Zn(II) complexes often exhibit a simplified Q-band spectrum, altered fluorescence, improved geometric organization of the macrocycle, and greater efficiency in the formation of a long-lived triplet state [6,17,22]. At the same time, Zn(II) does not produce such a pronounced heavy-atom effect as Pd(II) or Pt(II), and does not usually participate in the physiological redox cycling characteristic of Fe-, Mn- or Cu-porphyrins [75,214]. This places them in an intermediate but very important position: Zn-porphyrins are not the ‘heaviest’ photosensitizers, yet they often provide sufficiently efficient singlet oxygen generation whilst carrying a lower risk of metal-dependent dark toxicity.

A comparison with Pd- and Pt-porphyrins also reveals a trade-off between photoreactivity and translational safety. Heavy metals can dramatically enhance ISC and phosphorescence, which is useful for oxygen sensing and PDT [75]. However, such systems may have different toxicity profiles, more complex metabolism and higher requirements for pharmacokinetic control. Zn(II) porphyrins are less radical in terms of the heavy-atom effect, but it is precisely this that makes them suitable for a theranostic balance: fluorescent imaging, photodynamic action and relative chemical predictability can be combined within a single platform [7,24].

This intermediate position is best exemplified by the balance between singlet oxygen and radical photochemistry.

### 15.3. Balance of Type II and Type I Mechanisms as a Practical Limit of Zn-Porphyrin PDT

The main mechanism of Zn-porphyrin PDT is usually attributed to type II photochemistry, i.e., the transfer of energy from the triplet state of the photosensitizer to molecular oxygen, resulting in the formation of singlet oxygen [6,17]. This is consistent with the redox inertness of Zn(II) and the prominent role of the triplet state. However, this interpretation should not be oversimplified. In real cells, PDT rarely occurs as a pure type II process. Local oxygen concentration, the presence of reducing agents, lipids, proteins, NAD(P)H-dependent systems, membrane localization and aggregation of the photosensitizer can shift the mechanism towards Type I reactions involving the formation of superoxide, hydrogen peroxide, hydroxyl radicals or lipid radicals [12,39,124]. For Zn-porphyrins, this means that their typical Type II profile is not an absolute property of the molecule but rather the result of the interaction between photophysics and the specific microenvironment.

The practical significance of this balance is particularly evident in hypoxic tumors. If therapy relies entirely on singlet oxygen, a reduction in the partial pressure of O_2_ rapidly limits its efficacy. Therefore, Zn-porphyrin systems in which the molecular design or formulation partially supports the Type I component, facilitates oxygen diffusion, reduces aggregation, or is combined with hypoxia-modulating carriers are promising [16,122,169,174]. In this sense, the challenge lies not in completely replacing the Type II mechanism but in creating a more stable mixed photochemical profile.

However, the type of ROS does not explain the PDT outcome without taking into account the subcellular localization of the photosensitizer.

### 15.4. Subcellular Localization as a Key to Understanding Different Types of Cell Death

An important conclusion from the previous sections is that the toxicity of PDT is determined not only by the quantity of ROS but also by the site of their formation. Singlet oxygen has a short lifetime and a short effective diffusion distance in biological media; therefore the localization of Zn-porphyrin in mitochondria, lysosomes, the endoplasmic reticulum, the plasma membrane or near the nucleus significantly alters the effects of irradiation [12,156]. Mitochondrial localization promotes membrane depolarization, the release of cytochrome c, disruption of the respiratory chain and apoptosis [13,156]. Lysosomal localization can lead to lysosomal membrane permeability, the release of cathepsins, and a shift toward apoptosis or necrotic forms of cell death [160]. Damage to the ER induces oxidative stress, protein denaturation, activation of the UPR, calcium dysregulation and, in cases of severe damage, an apoptotic response [13,37]. Therefore, the same Zn-porphyrin may exhibit different biological effects depending on its charge, amphiphilicity, formulation and protein transport.

This explains why simple measures of ‘total ROS’ are of limited informative value. Two compounds may produce a similar DCFH-DA signal, but one will predominantly trigger mitochondrial apoptosis, while the other will induce lysosomal dysfunction or membrane necrosis. Consequently, a mechanistic characterization of Zn-porphyrin PDT should include confocal microscopy, colocalization with organelle markers, analysis of membrane potential, lipid peroxidation, caspases, LDH release and long-term clonogenic survival [12,96,156].

Localization identifies the primary targets, but the final phenotype is determined by the transition from local oxidation to a systemic cellular response.

### 15.5. Biochemical Damage: From Local Oxidation to Systemic Cellular Response

The biochemical mechanism of action of Zn-porphyrin PDT involves protein oxidation, lipid peroxidation, mitochondrial dysfunction, endoplasmic reticulum (ER) stress, activation of the proteasome, autophagy and secondary DNA responses. These processes are not independent of one another. The oxidation of cysteine, methionine, histidine, tryptophan and tyrosine can alter the activity of enzymes, receptors, transporters and cytoskeletal proteins [12,13]. Lipid peroxidation disrupts membrane integrity, forms reactive aldehydes and may trigger ferroptosis-like programs [39,126]. Mitochondrial damage amplifies the secondary ROS signal, reduces ATP levels and shifts the cell from adaptive stress to irreversible cell death [13,156]. Autophagy in this context is two-fold: under moderate stress, it can remove damaged proteins and organelles, but under severe damage, it becomes part of the lethal response or a marker of the cell’s inability to restore homeostasis [93].

Therefore, the biochemical effect of Zn-porphyrin PDT should be assessed as a temporal sequence. Early events include the photo-oxidation of the immediate target molecules; intermediate events involve the activation of stress pathways; and late events involve a choice between repair, apoptosis, necrosis, ferroptosis or immunogenic cell death. This approach is useful for experimental design: some markers need to be measured minutes after irradiation, others—hours or a day later [13]. Without temporal resolution, secondary adaptive responses may be mistakenly interpreted as primary mechanisms of photodynamic action.

This is precisely why the modern concept of PDT must include not only apoptosis or necrosis, but also ferroptosis-like and immunogenic response variants.

### 15.6. Ferroptosis, Immunogenic Death and the Extension of the Classical PDT Concept

The current understanding of PDT goes beyond the simple ‘ROS—necrosis or apoptosis’ model. Ferroptosis-like mechanisms and immunogenic cell death are particularly important. Although Zn(II) is not an iron-like redox-active center, Zn-porphyrin PDT may indirectly enhance ferroptosis via lipid peroxidation, depletion of antioxidant systems, disruption of the glutathione buffer, or functional inhibition of GPX4-dependent defense [39,127,216]. In turn, immunogenic cell death links local photo-oxidative damage to a systemic anti-tumor response: exposure to calreticulin, the release of ATP, HMGB1 and heat shock proteins, and the activation of dendritic cells [115,173]. This is particularly promising for metal porphyrins, as the central metal can indirectly influence the intensity of stress, the localization of damage and the profile of DAMP signals.

These areas are of not only fundamental but also therapeutic significance. Combining PDT with ferroptosis inducers, inhibitors of antioxidant systems, immune checkpoint blockade or immunomodulatory adjuvants could transform local phototherapy into a systemically significant anti-tumor strategy [39,173,217]. However, such combinations require caution, as increased oxidative stress may also exacerbate damage to normal tissues if there is insufficient selectivity in the accumulation of the photosensitizer.

Expanding the mechanistic concept highlights the role of formulations capable of modifying not only the delivery but also the very mode of photodynamic action.

### 15.7. Formulations and Nanocarriers as a Way to Change the Mechanism, Not Just the Delivery

Nanoparticle formulations are often regarded as a technical solution to the problems of solubility and selective delivery, but in the case of Zn-porphyrin PDT, they should be viewed in a broader context. Liposomes, micelles, polymeric nanoparticles, protein carriers, peptide conjugates, MOFs and coordination polymers alter the aggregation state, oxygen availability, microlocalization within the cell, and the kinetics of photosensitizer release [16,24,173,178,198]. Particularly noteworthy are porphyrin-based MOF platforms, in which porphyrin ligands can simultaneously act as a structural element, a photosensitizer and a functional hub for combined PDT, catalytic therapy, ferroptosis or hypoxia-modulating strategies [16,169,172]. Consequently, the formulation does not merely ‘deliver’ Zn-porphyrin to the tumor but effectively sets the spatial and chemical conditions under which its photochemistry takes place. More information about the influence of formulation of Me-porphyrins is presented in Table 3.

This is particularly relevant for hydrophobic Zn-porphyrins, which are prone to aggregation-induced quenching. In a poorly designed formulation, even a photophysically promising molecule may lose its activity due to self-quenching, poor cellular penetration, or unfavorable binding to serum proteins [24,198]. Conversely, a properly selected carrier can increase the local concentration of the monomeric form, direct the photosensitizer to the mitochondria or lysosomes, and thereby alter not only the strength but also the type of cellular response.

Nevertheless, potent photochemistry is only of value when combined with clinical controllability and acceptable safety.

### 15.8. Translational Limitations: Between Strong Photochemistry and Clinical Manageability

The clinical prospects for Zn-porphyrins depend on the extent to which their advantages can be translated into a reproducible therapeutic effect. Strong generation of ROS is not a sufficient condition for successful PDT. Controlled dark toxicity, predictable pharmacokinetics, low systemic photosensitivity, a stable formulation, acceptable clearance, reproducible synthesis, and standardized light dosimetry are required [7,24,102]. Furthermore, the tumor microenvironment imposes significant constraints: hypoxia, acidity, heterogeneous vascularization, a dense extracellular matrix, and binding to albumin or lipoproteins can alter the actual concentration of the active photosensitizer in tumor tissue [16,102,218]. Therefore, the future translation of Zn-porphyrin systems requires not only optimization of the molecule but also comprehensive validation of the ‘molecule–carrier–light dose–tissue oxygen–biological response’ pathway.

A particular challenge lies in the standardization of preclinical models. In vitro results are often obtained under conditions that do not reflect the actual tissue thickness, diffusion barriers, oxygen gradient and the influence of the immune system. Therefore, promising Zn-porphyrin systems must be tested not only in monolayer cultures but also in spheroids, organoids, endothelial models, immunocompetent animal systems, and models with controlled oxygenation [7,24,102]. This will make it possible to distinguish a truly translational photosensitizer from a molecule that is active only in an optimized laboratory system.

This leads to the main point of discussion: the future development of Zn-porphyrin PDT must be mechanism-driven. Zn(II) porphyrins, traditionally Type II sensitizers, can be engineered to generate Type I reactive oxygen species under hypoxia via structural modifications like halogenation or cationic substitution. These modifications reduce LUMO levels and facilitate electron transfer from intracellular reductants (e.g., NADH) to generate cytotoxic radicals, as demonstrated in recent studies involving mitochondria-targeted and nanoformulated systems.

### 15.9. Mechanism-Driven Design as Pre-Conclusion of the Review

The overall conclusion of the discussion is that the future development of metal porphyrin PDT should move away from the empirical selection of photosensitisers towards mechanism-driven design. The central metal, peripheral substituents, charge, lipophilicity, axial ligands, nanocarriers, organelle targeting and light dose should be considered as interrelated parameters of a single system [24,218,219,220]. For Zn(II) porphyrins, the greatest strength lies in the balance between photodynamic activity, fluorescence suitability, theragnostic potential and relative redox safety. Their main limitations remain their dependence on oxygen, the risk of aggregation, insufficient selectivity without carriers, and the need for precise control of localization. Therefore, Zn-porphyrins should be regarded not as universal photosensitizers, but as a flexible platform, the effectiveness of which is determined by a rational combination of metal-centered photophysics, biochemical context and translational engineering.

Ultimately, the central metal is not merely a chemical modification of the porphyrin but a regulator of the cause-and-effect chain of PDT. For Zn(II) porphyrins, this chain can be formulated as follows: Zn(II) coordination alters the electronic structure and photostates; the photostats determine the profile of ROS; ROS in a specific organelle trigger specific biochemical damage; the damage determines the type of cell death; and the tissue microenvironment and formulation determine whether this response will be therapeutically beneficial. It is precisely this integrative model that should form the basis for the future design of Zn-porphyrin photosensitizers [7].

After a debatable generalization, we can proceed to concise conclusions that record the main structural, photophysical, biochemical, and translational provisions of the review. To clarify the precise origin of the photophysical advantages in these systems, it is essential to isolate the electronic contributions of the central Zn(II) ion from the structural influences of the surrounding macrocycle. While the porphyrin scaffold, peripheral substituents, and aggregation state dictate tissue-level variables—such as solubility, amphiphilicity, and targeted subcellular localization—the central metal ion fundamentally governs the core photophysical transitions of the macrocycle.

Systematic comparative studies utilizing identical porphyrin scaffolds coordinated to different central metal ions demonstrate that the insertion of Zn(II) provides a unique, intrinsic advantage that cannot be replicated by the macrocycle alone.

## 16. Conclusions

The central metal ion is a critical determinant of a porphyrin’s electronic structure, light absorption profile, triplet state transition efficiency, and overall cellular response. Consequently, PDT mechanisms must be viewed as an integrated result of interactions between the metal center, peripheral substituents, delivery formulation, microenvironment, and biological target.

Within this framework, Zn(II) porphyrins offer a unique advantage due to their closed d10 electronic configuration. Unlike redox-active metals like iron, manganese, or copper, which catalyze unpredictable, uncontrolled redox processes, zinc does not undergo intense redox cycling under physiological conditions. This allows Zn(II) porphyrins to combine high photochemical activity with favorable biocompatibility. They serve as excellent models for Type II PDT, where energy transfer from the triplet state to molecular oxygen, generating singlet oxygen, is the dominant pathway. Furthermore, the zinc ion alters the symmetry of the porphyrin core to optimize absorption, fluorescence, photostability, and triplet yield. This dual capacity for photodynamic activity and optical informativeness enables Zn(II) porphyrins to serve as advanced theranostic platforms for simultaneous tumor imaging, biodistribution tracking, and therapy.

The ultimate therapeutic outcome relies heavily on the specific subcellular localization of the photosensitizer, as the same agent can trigger different cell death pathways depending on where it accumulates. Mitochondrial accumulation induces membrane depolarization, cytochrome c release, and apoptosis. Lysosomal localization causes membrane permeability and cathepsin release, while damage to the endoplasmic reticulum disrupts calcium homeostasis and triggers misfolded protein responses. Meanwhile, targeting the plasma membrane or vascular endothelium shifts the biological response toward necrosis or vascular collapse. Equally vital is the advanced formulation, which dictates whether the photophysical potential of the metal center is realized in vivo. Because hydrophobicity causes aggregation and self-quenching, the use of liposomes, micelles, nanoparticles, and organelle-targeted carriers is essential to preserve solubility and ensure stable biodistribution. Additionally, tumor hypoxia remains a strict limiting factor for these heavily oxygen-dependent Type II photosensitizers. To overcome this, next-generation Zn(II) systems must incorporate strategies that enhance Type I mechanisms, deliver oxygen, or combine PDT with complementary treatments like immunotherapy, chemotherapy, ferroptosis inducers, or nanocatalytic systems. Ultimately, shifting from empirical use to the rational, mechanism-driven design of next-generation PDT agents requires balancing these interconnected systems of chemistry, formulation, and biological context. Accurately predicting the in vivo therapeutic outcomes of Zn(II) porphyrin photodynamic therapy requires evaluating photophysical properties, such as singlet oxygen quantum yield, in conjunction with physiological parameters like aqueous aggregation behavior and subcellular localization. While hydrophobic Zn(II) porphyrins may offer superior theoretical efficiency in solvent, amphiphilic or cationic variants often demonstrate better efficacy in vivo by preventing self-aggregation and optimizing targeted delivery to critical cellular compartments.

Future Directions: The transition from empirical utilization of porphyrins to the rational, mechanism-driven design of next-generation PDT agents requires a concerted focus on several key areas. Since the Zn(II) ion establishes a highly favorable balance between photophysical efficiency and biological compatibility, future research should leverage this unique center to develop advanced theragnostic platforms. To fully realize this potential, future structural modifications must focus on altering the symmetry of porphyrin core and optimizing peripheral substituents. This structural engineering should aim to fine-tune absorption bands, maximize triplet yields, and intentionally enhance Type I photochemical mechanisms to ensure therapeutic efficacy even within severely hypoxic tumor microenvironments. Simultaneously, the development of advanced formulations must progress in tandem with chemical synthesis. Future studies should prioritize the integration of Zn(II) porphyrins into smart nanocarriers—such as liposomes, polymeric micelles, metal–organic frameworks, and organelle-targeted peptide conjugates—to systematically prevent self-quenching, control aggregation, and dictate precise subcellular localization. By guiding the photosensitizer to specific targets like the mitochondria or endoplasmic reticulum, therapies can deliberately trigger desired cell death pathways. Finally, future translational strategies should exploit the intrinsic optical informativeness of these systems for real-time fluorescence imaging and treatment monitoring, while combining Zn(II) PDT with complementary modalities such as immunotherapy, chemotherapy, and ferroptosis inducers to overcome tissue resistance and achieve systemic anti-tumor efficacy. While the d10 electronic configuration of Zn(II) porphyrins consistently boosts triplet-state formation and singlet oxygen ^1^O_2_ generation compared to free-base porphyrins, these superior photophysical properties do not always translate into enhanced PDT efficacy. Comparative studies indicate that while Zn(II) porphyrins outperform heavy metal analogs (Pd, Pt) in tumor cell killing due to lower dark toxicity, their in vivo success is ultimately dependent on a balance of cellular uptake and localization rather than high ROS generation alone. Detailed comparative studies on in vitro and in vivo efficacy are required for specific applications.

## Figures and Tables

**Table 1:** Metal-dependent regulation of photophysical properties of porphyrin photosensitizers in PDT.

Central Metal/System	Electronic Character	Effect on Soret/Q Bands	Fluorescence	ISC/Triplet State	Typical ROS Profile	Practical Relevance for PDT
Metal-free porphyrins [36,44]	No central metal; two inner N-H protons; lower macrocycle symmetry [42,43]	Intense Soret band; usually four weaker Q bands because of reduced symmetry [42,71]	Usually detectable; may be useful for imaging depending on substituents [44,46]	Moderate ISC; triplet yield depends strongly on macrocycle substitution, aggregation and medium [53,72]	Mainly Type II singlet oxygen under oxygenated conditions; Type I possible in specific environments [6,8]	Reference platform for structure-activity comparison; useful when strong metal-related effects are not desired [15,44]
Zn(II) porphyrins [17,22]	Closed-shell d10 Zn(II); generally redox-inert under physiological conditions [14,30]	Symmetrization of the macrocycle; usually simplified Q-band pattern; ligand-centered π-π* transitions dominate [42,46]	Often preserved or enhanced compared with paramagnetic metal complexes [46,73]	Favorable balance between fluorescence and triplet formation; efficient oxygen sensitization in many derivatives [17]	Predominantly Type II singlet oxygen; secondary ROS arise from biomolecular oxidation [6,17]	Key theranostic class: combines fluorescence, PDT activity, relative biocompatibility and coordination sensitivity [16,22]
Mg(II)-porphyrins/chlorophyll-like systems [32,36]	Main-group Mg(II); biologically relevant in chlorophylls; not strongly redox-cycling [32,45]	Chlorophyll-like absorption when macrocycle is reduced to chlorin; synthetic Mg-porphyrins may be chemically labile [36,45]	Variable; strongly affected by demetallation, aggregation and environment [36,45]	Excited-state behavior can be productive, but chemical stability may limit use [30,32]	Singlet oxygen generation possible; performance depends on macrocycle and formulation [6,35]	Useful model systems and inspiration for chlorin-type photosensitizers; less robust than many Zn complexes [32,44]
Pd(II)- and Pt(II)-porphyrins [32,52]	Heavy d8 metal centers; strong spin–orbit coupling [32,52]	Soret/Q bands remain macrocycle-based but heavy-atom perturbation changes excited-state dynamics [30,52]	Often markedly quenched; phosphorescence may become important [32,52]	Very strong heavy-atom effect; high triplet yield and long-lived triplet states [32,52]	Efficient Type II singlet oxygen; Type I reactions can contribute depending on microenvironment [52,74]	High PDT potency and oxygen-sensing potential, but translational use requires careful toxicity and pharmacokinetic control [32,75]
Fe-, Mn- and Cu-porphyrins [7,76]	Open-shell or redox-active transition metals; multiple oxidation states possible [32,76]	Complex spectral changes; metal-centered states may contribute to non-radiative pathways [30,33]	Often quenched, especially for paramagnetic complexes [20,76]	Triplet states may be quenched or redirected through metal-centered states [20,76]	Greater Type I/radical contribution; catalytic redox chemistry may occur [74,76]	Mechanistically powerful but biologically complex; useful for hypoxia-oriented or redox-modulating strategies with safety constraints [76,77]

**Table 2:** Influence of central metal on metalloporphyrin localization.

Localization of Photosensitizer	Primary ROS-Sensitive Targets	Main Biochemical Events	Probable Cellular Outcome	Experimental Markers/Assays
Mitochondria [79,83]	Cardiolipin, respiratory-chain complexes, outer mitochondrial membrane proteins, BCL-2 family regulators [72,80]	Membrane depolarization, mitochondrial outer membrane permeabilization, cytochrome c release, caspase cascade activation [37,80]	Intrinsic apoptosis; at high light doses, mixed apoptosis-necrosis [37,94]	JC-1/TMRE for Δψm; cytochrome c release; caspase-9/-3 activation; PARP cleavage; Annexin V/PI [37,81]
Lysosomes [79,81]	Lysosomal membrane lipids and proteins, cathepsins, endocytic cargo [80,95]	Lysosomal membrane permeabilization, cathepsin release, secondary mitochondrial injury, autophagic stress [80,96]	Apoptosis, necrosis, autophagy-associated death or mixed phenotypes [95,97]	LysoTracker loss; acridine orange relocation; cathepsin B/D release; LC3-II and p62 dynamics [96,97]
Endoplasmic reticulum [83]	Folding enzymes, chaperones, disulfide-bond systems, Ca^2+^-handling proteins [9,72]	Protein oxidation, unfolded protein response, Ca^2+^ dysregulation, CHOP induction [9,72]	Adaptive ER stress at low dose; apoptosis or immunogenic stress at higher dose [94,98]	PERK/eIF2alpha, IRE1/XBP1, ATF6, BiP/GRP78, CHOP, cytosolic Ca^2+^ measurements [9,72]
Plasma membrane [72,80]	Unsaturated lipids, receptors, transporters, membrane cytoskeleton [72,99]	Lipid peroxidation, permeability changes, receptor dysfunction, ionic imbalance [72,99]	Necrosis, necroptosis-like response or rapid membrane failure [96,99]	Propidium iodide uptake; LDH release; lipid peroxidation probes; MLKL/RIPK1 when necroptosis is suspected [100,101]
Nucleus/perinuclear region [72,83]	DNA bases, nuclear proteins, chromatin-associated proteins [72]	Oxidative DNA damage, replication stress, DNA repair signaling [72,100]	Genotoxic stress; apoptosis if repair capacity is exceeded [72,96]	gammaH2AX, 8-oxo-dG, comet assay, p53 pathway markers, cell-cycle analysis [100,101]
Tumor endothelium/vascular compartment [102,103]	Endothelial membranes, adhesion proteins, vasoactive signaling proteins [102,103]	Endothelial damage, platelet adhesion, vascular shutdown, ischemia of tumor tissue [102,103]	Secondary tumor cell death through vascular collapse and hypoxia [102,103]	CD31 staining, perfusion assays, vascular leakage, thrombosis markers, oxygenation measurements [102]
Microbial envelope/biofilm matrix [90,104]	Bacterial membranes, cell-wall components, extracellular polymeric substances [90,105]	Envelope oxidation, loss of membrane potential, biofilm matrix disruption [90,104]	Photodynamic inactivation of bacteria or fungi; biofilm weakening [90,105]	CFU counts, live/dead staining, crystal violet biofilm assay, membrane-potential probes [90,105]

**Table 3:** Formulation and translational strategies for improving Zn(II) porphyrin-mediated PDT.

Strategy	Main Problem Addressed	Mechanistic Advantage	Potential Limitations	Best-Suited Applications
Liposomes [28,203]	Poor aqueous solubility; aggregation of hydrophobic Zn-porphyrins [28,203]	Membrane-like environment stabilizes monomeric photosensitizer, improves light absorption and singlet-oxygen generation [28,73]	Batch-to-batch variability; leakage; size and stability control [28,203]	Dermatological PDT, local tumor PDT, membrane-associated delivery [28,193]
Polymeric micelles [198,218]	Hydrophobicity and rapid clearance [198,218]	Hydrophobic core solubilizes porphyrins; hydrophilic corona improves colloidal stability and circulation [198,218]	Premature release; polymer biocompatibility; dilution instability [198,218]	Systemic delivery of hydrophobic Zn-porphyrins [198,218]
Albumin-binding systems [177,203]	Need for biocompatible blood transport [177,203]	Uses endogenous protein transport; may improve tumor accumulation and reduce nonspecific aggregation [180,203]	Variable binding affinity; competition with endogenous ligands [177,203]	Soft formulations for translationally oriented PDT [177,203]
Inorganic or hybrid nanoparticles [28,202]	Need for multifunctional delivery and imaging [28,201]	Controlled loading, targeting ligands, combined PDT/PTT/chemotherapy/immunotherapy and imaging [28,201]	Carrier toxicity; long-term fate; regulatory complexity [28,205]	Theranostics and combination therapy [177,219]
Mitochondria-targeted Zn-porphyrins [80,203]	Need for strong apoptotic response [80,203]	Cationic or lipophilic groups exploit mitochondrial membrane potential and localize ROS near apoptotic machinery [80,203]	Possible dark toxicity; selectivity must be validated [80,203]	Cancer cells with high mitochondrial vulnerability [80,203]
Lysosome-targeted delivery [80,83]	Need to trigger LMP and endocytic escape [81,96]	Endocytic uptake directs photosensitizer to lysosomes; ROS release cathepsins and may amplify death signaling [81,96]	Mixed death phenotypes; risk of inflammation [96,97]	Tumors resistant to classic mitochondrial apoptosis [96,220]
Oxygen-supplying or oxygen-generating systems [150,151]	Hypoxia and rapid oxygen consumption during PDT [150,151]	Perfluorocarbon carriers, catalase-like particles or H_2_O_2_-responsive systems support Type II photochemistry [150,151]	Oxygen control is difficult; safety and kinetics must be optimized [150,151]	Hypoxic tumors and thick lesions [154,156]
Type I-oriented chemical modification [67,69]	Reduced efficiency of Type II PDT at low oxygen tension [67,69]	Electron-transfer or radical-generating design may maintain phototoxicity under limited oxygen [67,208]	More complex ROS biology; higher risk of off-target radical damage [67,69]	Hypoxic tumors, biofilms, low-oxygen microenvironments [67,74]
Receptor-targeted systems [168,210]	Need for cellular selectivity [168,210]	Folate, RGD peptides, antibodies or aptamers can enhance accumulation in target cells [168,210]	Receptor heterogeneity; immunogenicity; manufacturing complexity [168,210]	Precision PDT and tumor-selective accumulation [168,211]
Antimicrobial cationic Zn-porphyrins [25,104]	Microbial membranes and biofilms are difficult to eradicate [89,105]	Positive charge enhances binding to bacterial envelopes and biofilm matrices; local ROS bypasses classic antibiotic resistance [25,104]	Host–cell selectivity and light delivery must be controlled [89,105]	Antimicrobial PDT, wound disinfection, biofilm-associated infections [90,104]

## Data Availability

No new data were created or analyzed in this study. Data sharing is not applicable to this article.
